# New Evidence in the Booming Field of Online Mindfulness: An Updated Meta-analysis of Randomized Controlled Trials

**DOI:** 10.2196/28168

**Published:** 2021-07-19

**Authors:** Marion Sommers-Spijkerman, Judith Austin, Ernst Bohlmeijer, Wendy Pots

**Affiliations:** 1 Department of Rehabilitation, Physical Therapy Science and Sports University Medical Center Utrecht Utrecht Netherlands; 2 Department of Psychology, Health and Technology University of Twente Enschede Netherlands

**Keywords:** mindfulness, mental health, intervention, online, meta-analysis, mobile phone

## Abstract

**Background:**

There is a need to regularly update the evidence base on the effectiveness of online mindfulness-based interventions (MBIs), especially considering how fast this field is growing and developing.

**Objective:**

This study presents an updated meta-analysis of randomized controlled trials assessing the effects of online MBIs on mental health and the potential moderators of these effects.

**Methods:**

We conducted a systematic literature search in PsycINFO, PubMed, and Web of Science up to December 4, 2020, and included 97 trials, totaling 125 comparisons. Pre-to-post and pre-to-follow-up between-group effect sizes (Hedges *g*) were calculated for depression, anxiety, stress, well-being, and mindfulness using a random effects model.

**Results:**

The findings revealed statistically significant moderate pre-to-post effects on depression (*g*=0.34, 95% CI 0.18-0.50; *P*<.001), stress (*g*=0.44, 95% CI 0.32-0.55; *P*<.001), and mindfulness (*g*=0.40, 95% CI 0.30-0.50; *P*<.001) and small effects on anxiety (*g*=0.26, 95% CI 0.18-0.33; *P*<.001). For well-being, a significant small effect was found only when omitting outliers (*g*=0.22, 95% CI 0.15-0.29; *P*<.001) or low-quality studies (*g*=0.26, 95% CI 0.12-0.41; *P*<.001). Significant but small follow-up effects were found for depression (*g*=0.25, 95% CI 0.12-0.38) and anxiety (*g*=0.23, 95% CI 0.13-0.32). Subgroup analyses revealed that online MBIs resulted in higher effect sizes for stress when offered with guidance. In terms of stress and mindfulness, studies that used inactive control conditions yielded larger effects. For anxiety, populations with psychological symptoms had higher effect sizes. Adherence rates for the interventions ranged from 35% to 92%, but most studies lacked clear definitions or cut-offs.

**Conclusions:**

Our findings not only demonstrate that online MBIs are booming but also corroborate previous findings that online MBIs are beneficial for improving mental health outcomes in a broad range of populations. To advance the field of online MBIs, future trials should pay specific attention to methodological quality, adherence, and long-term follow-up measurements.

## Introduction

### Background

In the 1970s, mindfulness was introduced as an intervention to tackle various psychological symptoms, such as stress, depression, and anxiety [1]. Mindfulness has been defined as the ability to be aware of bodily sensations, feelings, and thoughts in the present moment with a curious and accepting attitude toward these experiences [2,3]. Since the 1970s, various mindfulness-based interventions (MBIs) have been developed. Mindfulness-Based Stress Reduction (MBSR) was originally developed for people with chronic pain to support them in coping with chronic stress [2,4]; Mindfulness-Based Cognitive Therapy (MBCT) was primarily developed for people with recurrent depression [5]; Dialectical Behavior Therapy was developed for cultivating emotion-regulation skills in people with borderline personality disorder [6]; and Acceptance and Commitment Therapy (ACT) was developed to increase psychological flexibility and reduce distress in people with various mental health problems [7]. Although these interventions vary in the use and duration of specific meditation exercises and in their theoretical and psycho-educational frameworks, they share a core focus on promoting awareness of sensations, emotions, and cognitions and the ability to not react to and identify with these bodily and mental events. Over the past decades, a large number of systematic reviews and meta-analyses have been conducted on the effectiveness of MBIs in various target groups [8-24]. Following a general trend in mental health care, MBIs are increasingly being delivered through the internet [25-27]. In 2015, we conducted a meta-analysis to examine the effectiveness of online MBIs published in 2016 [28]. In this meta-analysis, 15 randomized controlled trials (RCTs) totaling 17 comparisons of an online MBI with a control group were included. At postintervention, online MBIs outperformed controls on all outcomes. Short-term effects were promising and included enhanced well-being (*g*=0.23), decreased depressive and anxiety symptomatology (*g*=0.29 and *g*=0.22, respectively), reduced levels of stress (*g*=0.51), and improved mindfulness (*g*=0.32). Although these findings indicate that online MBIs have the potential to contribute toward improving mental health, the observed effects had to be interpreted with caution considering the limited number of included studies and the fact that many of the included RCTs were limited in scope, comparability, and methodological quality.

One year later, another meta-analysis focused on the same topic [10]. This meta-analysis included data collected until October 23, 2015, and showed considerable overlap with our meta-analysis; 21 RCTs were included in this study. The findings indicated significant pre-post improvements in anxiety, depression, and quality of life compared with the control conditions. Online MBIs were not found to be more effective than the comparison interventions.

In 2018, Sevilla-Llewellyn-Jones et al [9] performed a systematic review and meta-analysis on the effectiveness of online MBIs, specifically focusing on populations with diagnosed mental health problems. On the basis of 12 studies, online MBIs were found to significantly improve mindfulness skills in people with mental disorders. Furthermore, the findings demonstrated that online MBIs are effective in reducing depression and anxiety and improving quality of life in people with anxiety disorder, but not in people with depressive disorder.

In 2020, two more meta-analyses of technology-enabled and online MBIs were performed, focusing on stress management in the general population (n=16) [29] and distress in clinical and nonclinical populations (n=43) [30]. The findings demonstrated small-to-medium effects on stress, anxiety, depression, and mindfulness compared with active and nonactive control conditions [29,30]. As these reviews address a narrower target population [29] and scope of interventions [30] than our original meta-analysis, a comprehensive review of online MBIs is still lacking.

### Objectives

Indeed, reviewing the literature from the past few years indicates that developments in the area of online mindfulness emphasize the need to regularly update the current evidence base. First, as anticipated, the field of online mindfulness is booming, as evidenced by dozens of studies that have been published since our previous meta-analysis. Incorporating these studies in a new meta-analysis would provide a more thorough assessment of the clinical and nonclinical utility of online MBIs and improve the power of moderation analyses. Second, we noticed a transformation in the types of online MBIs that are being delivered to users. At the time that our 2016 meta-analysis was conducted, MBSR and MBCT and derivatives from these interventions dominated the field. Since then, there has been a rapid increase in online ACT interventions, allowing a more robust assessment of this specific MBI. Third, it appears that the components of MBIs are increasingly mixed or hybrid, resulting in numerous MBIs that show considerable overlap and all seem to have a beneficial impact on mental health. It remains unclear whether mixtures of MBIs are equally effective in improving mental health. Fourth, not only is the content of today’s online MBIs different from 5 years ago but also the delivery method is different. MBIs are increasingly delivered through smartphone apps instead of websites, increasing access modality and ease of usage. These developments spurred the desire to update and extend our 2016 meta-analysis to provide more robust evidence of the short-term and long-term effects of online MBIs, as well as the potential moderators of these effects.

## Methods

This study was conducted according to the PRISMA (Preferred Reporting Items for Systematic Reviews and Meta-Analyses) guidelines [31]. The data collection and analysis procedures were similar to those used in our previous meta-analysis [28].

### Literature Search and Eligibility Criteria

We searched the PsycINFO, PubMed, and Web of Science databases three times for RCTs published since 2015—that is, September 6, 2018; July 19, 2019 (by MSS and WP); and December 4, 2020 (by JA). The search strategy was identical to that used in our previous meta-analysis [28]. Search terms included synonyms, both in text words and Medical Subject Headings or thesaurus terms, for (1) mindfulness (eg, mindful* and *meditation*), (2) intervention (eg, intervention* and treatment*), (3) online (eg, *e-health* and Internet*), and (4) RCTs (eg, random* and *trial*). The search results were filtered for English-language journal articles. For complete search strings, we refer to our previous meta-analysis [28].

Trials were included when they met the following criteria:

- Examines the effectiveness of an MBI, that is, an intervention consisting of at least one guided or unguided session and a combination of psycho-education and more than one experiential exercise with a primary focus on enhancing mindfulness skills. Both mindfulness-only interventions (eg, MBSR and MBCT) and mindfulness-integrative interventions such as Dialectical Behavior Therapy, ACT, or Mindfulness-Based Compassionate Living were included. Derivatives and mixtures of programs were also eligible, provided that teaching mindfulness was at the core of the intervention.
- The MBI is delivered via the internet and can be followed on a computer or a mobile device such as a smartphone or tablet. Interventions that used a combination of face-to-face and online sessions were eligible when face-to-face sessions were limited to the introduction of the study.
- Use of a randomized controlled design with at least one experimental condition and one active or inactive control condition (ie, no treatment, usual care, or any active treatment other than the experimental intervention).
- Depressive symptoms, anxiety symptoms, stress, well-being, or mindfulness was measured pre- and postintervention, using a validated measure.
- Studies simultaneously using MBIs and non-MBIs were eligible for inclusion, provided that the design allowed us to distinguish the independent effects of the MBIs.
- The study population consisted of adults aged ≥18 years. Both clinical (mental and physical disorders) and nonclinical samples (eg, students and community samples) were eligible.
- The reported findings allow the calculation of effect sizes, or the necessary data were made available by the authors. In addition, RCT protocols were screened for eligibility and included when the authors provided the necessary data.

The selection of studies took place in three phases: first, the review of titles; second, abstracts; and third, full texts. The selection was conducted independently by MSS, JA, and WP. Disagreements were resolved through discussion.

### Data Extraction

Population, intervention, and methodological characteristics (Multimedia Appendix 1; Gao et al, unpublished data, 2021; [32-125]) as well as effect size data were extracted from the full-text papers by 3 raters (ie, MSS, JA, and WP) independently. When discrepancies occurred, these were resolved in the discussion. When the article provided insufficient information regarding the study characteristics, the authors were contacted.

### Quality Assessment

Three raters (alternatingly, MSS, JA, and WP) independently assessed the methodological quality of each study using the same criteria as outlined in [28]. In brief, the criteria included (1) adequate sequence generation and allocation concealment; (2) blinding of main outcome assessments; (3) drop-out analysis; (4) adequate handling of missing data; (5) adequate sample size calculation; (6) comparability of experimental and control participants at baseline; and (7) diagnostic assessment of participants (only applicable for clinical samples). Raters coded each criterion as 1 (criterion is met) or 0 (criterion is not met). Disagreements between raters were resolved through discussion. Studies were scored between 0 and 7 points, with higher scores reflecting greater methodological quality. The methodological quality of each study was assessed as high (7 points), moderate (5-6 points), or low (≤4 points).

### Data Analysis

Meta-analytic procedures were performed using the Comprehensive Meta-Analysis software, version 2.2.064. Hedges *g* effect sizes were calculated for (1) depressive symptoms, (2) anxiety symptoms, (3) stress, (4) well-being, and (5) mindfulness, using the same steps as reported in [28]. We calculated pre-to-post between-group effect sizes for all studies and pre-to-follow-up between-group effect sizes, thereby including only studies with a follow-up period of 1-3 months. Effect sizes reflect the number of SDs with which the online MBI group had more changed than the control condition between pre- and postmeasurement and pre- and follow-up measurement. Where available, intention-to-treat data were used to calculate effect sizes. If a study used more than one measure for the same outcome, we used the most valid instrument. However, in the case of well-being, we observed different dimensions of well-being (eg, a measure of emotional well-being and a measure of psychological well-being). In these cases, we extracted all relevant outcomes and computed the combined (average) effect sizes. When studies used more than one comparison condition, we used the strongest comparison to calculate the effect size. Following the study by Lipsey and Wilson [126], effect sizes from 0 to 0.32 were considered a small effect, 0.33 to 0.55 were considered a moderate effect, and 0.56 to 1.20 were considered a large effect.

Per outcome, forest plots of the pre-to-post effect sizes and pre-to-follow-up effect sizes were generated. A random effects model was used [127]. Meta-analyses were conducted, both including and excluding outliers. Outliers were identified through visual inspection of forest plots. In line with our previous meta-analysis [28], a study was deemed an outlier when its 95% CI fell outside the 95% CI of the overall mean effect size (on both sides). As a sensitivity analysis, meta-analyses were repeated, thereby omitting low-quality studies (including outliers).

The statistical procedures used to assess heterogeneity, publication bias, and moderators were identical to those used in our previous meta-analysis [28]. A priori, specified subgroup analyses were conducted to assess the differential effects of online MBIs based on (1) intervention type: MBSR, MBCT, ACT, or MBI (ie, mixture); (2) therapist guidance, with or without; (3) delivery mode: app or website; (4) population type: clinical or nonclinical; (5) type of symptoms: psychological, physical, or no symptoms; and (6) type of control: active (ie, treatment as usual, psycho-education, or other intervention) or inactive (ie, waitlist or no intervention). We conducted mixed effects analyses, thereby using a random effects model to pool studies within each subgroup and a fixed effect model to test whether effect sizes between subgroups significantly differed from one another. Only subgroups with five or more comparisons were reported. In addition to study quality and number of intervention sessions, mean age and proportion of females were included in the meta-regression analyses (mixed effects model and unrestricted maximum likelihood). Subgroup and meta-regression analyses were conducted, including outliers and only with pre-to-post data.

## Results

### Selection of Studies

**Figure 1:**
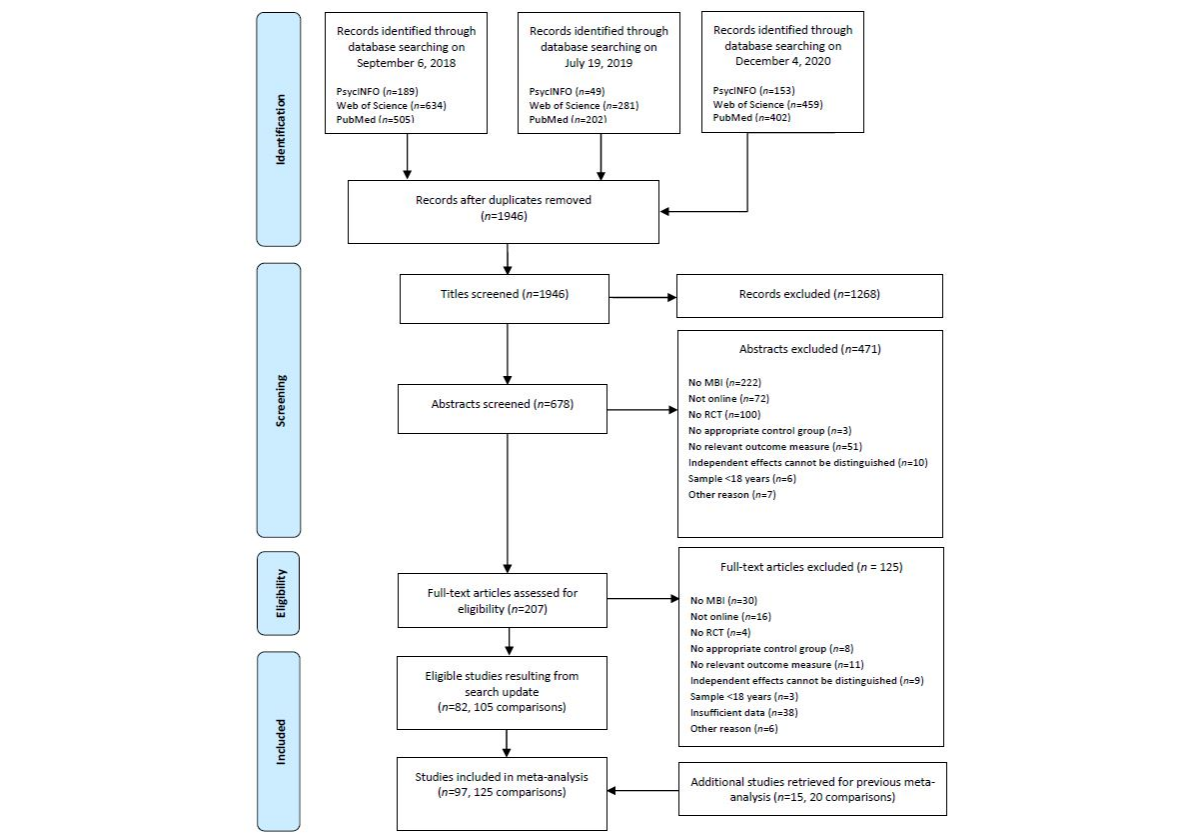
Flowchart of the study selection process. MBI: mindfulness-based intervention; RCT: randomized controlled trial.

The first search yielded 1328 hits, the second yielded 532 hits, and the last yielded 1014 hits. A total of 928 duplicates were removed. After reviewing 1946 titles, 678 abstracts, and 207 full articles, we identified 82 new studies, totaling 105 comparisons, which were not included in our previous meta-analysis (Figure 1). In addition, 15 eligible studies, including 20 comparisons identified in our previous meta-analysis [28], were included. Accordingly, 97 RCTs, totaling 125 comparisons, were included in this meta-analysis.

### Study Characteristics

Although research on the effectiveness of online MBIs has been undertaken in 21 countries across the globe, nearly one-third of all studies were conducted in the United States (n=31). Other countries in the top 5 included the United Kingdom, Sweden, the Netherlands, and China with 12, 10, 8, and 5 RCTs, respectively. Multimedia Appendix 1 shows the population, intervention, comparison, and outcome characteristics of all studies included in the meta-analysis.

The total study population consisted of 17,464 participants, with a mean age of 40 years. A total of 9066 participants were in the experimental condition and 7832 were in the control condition. There were large differences in sample sizes, ranging from 16 in a small-scale pilot RCT [32] to 2161 in a large-scale trial [33]. The proportion of women ranged from 41% to 100%. Most studies were conducted among the general population, employees, or student samples (45/97, 46% of studies). In 24 studies, online MBIs were targeted at populations with somatic illnesses, such as cancer (n=11) and chronic pain (n=7); 11 studies were targeted at health care professionals (n=4) and spousal or family caregivers (n=7). The remaining 17 studies included samples with psychological symptomatology, with depressive and anxiety symptoms being the most prevalent (n=12).

In 70.4% (88/125) of comparisons, a mindfulness-only intervention was used, with the most commonly studied intervention being MBSR (n=21), followed by MBCT (n=14), and a mixture or derivative of MBSR and MBCT and related exercises (n*=*53). Mindfulness-integrative interventions were used in the remaining 29.6% (37/125) of comparisons, specifically ACT (n=29), acceptance-based intervention (n=3), compassion-based intervention (n=4), and Mindfulness-Based Compassionate Living (n=1).

MBIs were mostly delivered through a website (n=84), followed by an app (n=27), virtual online classroom or videoconferencing software (n=4), or a combination (n=3). The number of online MBI sessions varied between 2 and 45. Sessions were used over a period of 10 days to 14 weeks. In 28.8% (36/125) of comparisons, online MBIs were provided with therapist guidance.

In 52% (65/125) of comparisons, the effectiveness of online MBIs was examined relative to a waitlist control (n=61) or no intervention (n=4) condition. An active control condition was used in 48% (60/125) of comparisons, including psycho-education (n=13), an online discussion forum (n=7), treatment as usual (n=14), and an alternative intervention (n=26; eg, expressive writing, cognitive behavioral therapy, and behavioral activation).

Outcome measures for depressive symptoms, anxiety, stress, well-being, and mindfulness were administered in 82, 70, 54, 48, and 67 comparisons, respectively; 44.3% (43/97) of studies reported not only pre- and postmeasurement but also follow-up measurements, with follow-up times ranging from 1 to 12 months.

### Adherence

Although 70% (68/97) of studies reported important information regarding adherence to the intervention (eg, time spent on the intervention, number of modules started, number of completed sessions, and daily meditation practice), only 23% (22/97) studies provided a definition or cut-off to determine adherence versus nonadherence. Using various definitions of adherence, these studies reported adherence rates ranging from 35% to 92%.

### Quality of Included Studies

**Figure 2:**
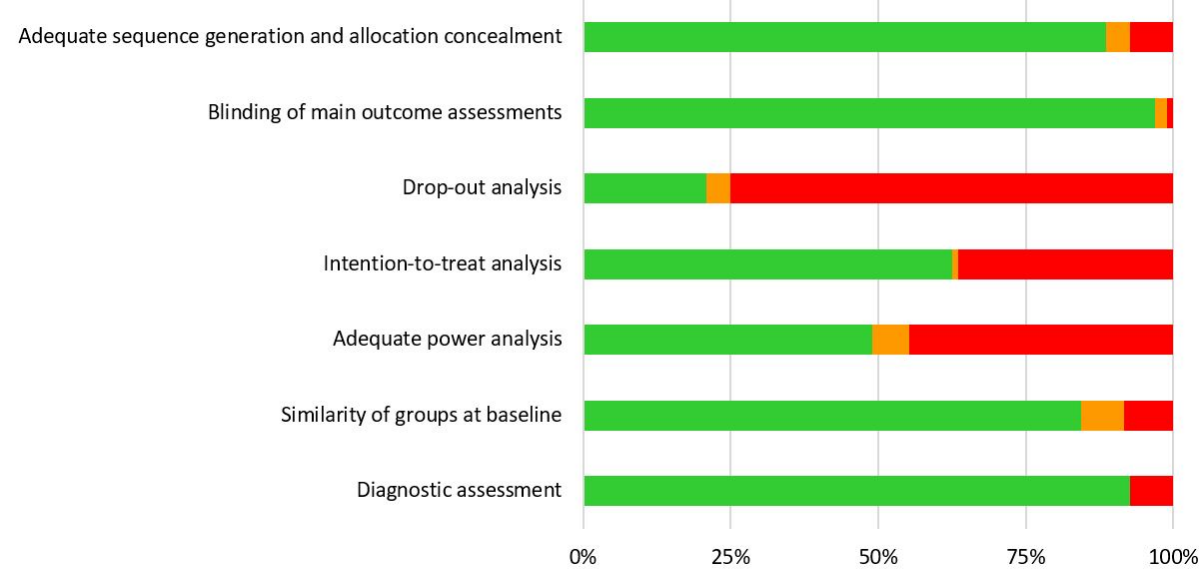
Results of methodological quality assessment per criterion presented as percentages across all included studies.

Scores for methodological quality varied between 1 and 7 points (Multimedia Appendix 2; Gao, M, unpublished data, 2021; [32-125]). Of the 97 included studies, 33 (34%) were considered low-quality studies; 66% (64/97) of the studies were rated as moderate (n=53) or high (n=11) quality. The results per quality criterion are shown in Figure 2.

### Pre-to-Post Between-Group Effects

#### Main Findings

Table 1 provides an overview of the pre-to-post between-group effects. For depression (89 comparisons) and anxiety (74 comparisons), significant moderate and small effects were observed (depression: *g*=0.34, 95% CI 0.18 to 0.50, *P*<.001; anxiety: *g*=0.26, 95% CI 0.18-0.33, *P*<.001). Significant, moderate effects were found for stress (56 comparisons; *g*=0.44, 95% CI 0.32-0.55, *P*<.001) and mindfulness (72 comparisons; *g*=0.40, 95% CI 0.30-0.50, *P*<.001). No significant effect was observed for well-being (52 comparisons; *g*=0.21, 95% CI –0.03 to 0.45, *P*=.08). The level of heterogeneity was moderate to high (*I^2^*=64.92-96.64). When outliers were removed, similar effect sizes were observed (depression: *g*=0.30; anxiety: *g*=0.22; stress: *g*=0.38; well-being: *g*=0.22; and mindfulness: *g*=0.39), with no changes in significance of the effect except for well-being (*P*<.001), and the level of heterogeneity remained moderate to high (*I^2^*=41.26-93.23). After omitting low-quality studies from the analysis, the effect size for well-being was significant (*g*=0.26, 95% CI 0.12-0.41; *P*<.001). For the remaining outcomes, no significant changes were observed for any of the outcomes, and all effect sizes remained virtually the same. Heterogeneity remained moderate to high (*I^2^*=65.72-83.06).

**Table 1:** Pre-to-post effects of online mindfulness-based interventions compared with controls^a^.

Outcomes		*N*_comp_^b^	Hedges *g* (95% CI)	*Z*	Heterogeneity		Fail-safe *N*
					*Q* value	*I* ^2^	
**All studies (including outliers)**							
	Depression	89	0.34 (0.18 to 0.50)	4.10^c^	1326.41^c^	93.37	5507
	Anxiety	74	0.26 (0.18 to 0.33)	6.70^c^	208.10^c^	64.92	2763
	Stress	56	0.44 (0.32 to 0.55)	7.48^c^	262.80^c^	79.07	1355
	Well-being	52	0.21 (–0.03 to 0.45)	1.75	1516.62^c^	96.64	121
	Mindfulness	72	0.40 (0.30 to 0.50)	7.72^c^	407.80^c^	82.59	2624
**All studies (excluding outliers)**							
	Depression^d^	86	0.30 (0.14 to 0.46)	3.69^c^	1254.96^c^	93.23	2903
	Anxiety^e^	67	0.22 (0.15 to 0.28)	6.62^c^	112.35^c^	41.26	1217
	Stress^f^	47	0.38 (0.29 to 0.48)	7.72^c^	130.94^c^	64.87	1947
	Well-being^g^	46	0.22 (0.15 to 0.29)	6.02^c^	89.18^c^	49.54	727
	Mindfulness^h^	65	0.39 (0.29 to 0.49)	7.65^c^	180.91^c^	74.37	3719
**Moderate- and high-quality studies**							
	Depression	61	0.37 (0.27 to 0.47)	7.43^c^	237.26^c^	74.71	2196
	Anxiety	55	0.28 (0.20 to 0.37)	6.35^c^	157.54^c^	65.72	1563
	Stress	38	0.39 (0.27 to 0.51)	6.26^c^	159.77^c^	76.84	1433
	Well-being	31	0.26 (0.12 to 0.41)	3.50^c^	154.25^c^	80.55	398
	Mindfulness	45	0.43 (0.29 to 0.56)	6.20^c^	259.72^c^	83.06	2486

^a^Analyses were conducted using a random effects model.

^b^*N*_comp_: number of comparisons.

^c^*P*<.001.

^d^Three outliers were removed: Kladnitski et al [34] (study 4); Querstret et al [35,36]; Yang et al [37].

^e^Seven outliers were removed: Forbes et al [38] (study 1); Gao, M (unpublished data, 2021); Levin et al [48]; Mak et al [39]; Querstret et al [35,36]; Segal et al [40]; Yang et al [37].

^f^Nine outliers were removed: Allexandre et al [41] (study 1); Beshai et al [42]; Champion et al [43]; El Morr et al [44]; Huberty et al [45]; Kladnitski et al [34] (study 4); Levin et al [46]; Nadler et al [128]; Walsh et al [47].

^g^Six outliers were removed: Allexandre et al [41] (study 1); Hoffmann et al [129]; Levin et al [130] (study 1); Levin et al [48]; Ly et al [49]; Mak et al [39].

^h^Seven outliers were removed: Forbes et al [38]; Henriksson et al [131]; Huberty et al [45]; Krusche et al [132]; Mak et al [33] (study 1 and study 2); Nadler et al [128].

#### Publication Bias

Whereas visual inspection of funnel plots indicated no remarkable evidence of publication bias, trim-and-fill analyses and fail-safe numbers suggest that publication bias has occurred in the reporting of effects on depressive and anxiety symptoms, well-being, and mindfulness. The Duval and Tweedie [133] trim-and-fill procedure indicated publication bias for depression, anxiety, and well-being. After adjustment for missing studies (n=19), the effect size for well-being dropped from *g*=0.21 to *g*=–0.04 (95% CI –0.23 to 0.15). For anxiety and depression, effect sizes were found to be higher, with three imputed studies on anxiety (*g*=0.31, 95% CI 0.23-0.38) and 19 on depression (*g*=0.48, 95% CI 0.39-0.57).

#### Subgroup Analyses

Subgroup analyses can be found in Multimedia Appendix 3. For stress, two subgroup analyses resulted in significantly higher effect sizes: (1) guided online MBIs compared with unguided online MBIs (guided: *g*=0.61, 95% CI 0.43-0.82; unguided: *g*=0.34, 95% CI 0.21-0.47; *P*=.02) and (2) studies that used an inactive control condition compared with studies with an active control group (inactive: *g*=0.56, 95% CI 0.43-0.69; active: *g*=0.15, 95% CI –0.04 to 0.35; *P*=.001). For mindfulness, inactive control groups also resulted in higher effect sizes than active control groups (inactive: *g*=0.52, 95% CI 0.41-0.63; active: *g*=0.19, 95% CI 0.03-0.34; *P*<.001). For anxiety, higher effect sizes were found in samples with psychological symptoms than in those with physical or no symptoms (psychological symptoms: *g*=0.47, 95% CI 0.33-0.61; physical symptoms: *g*=0.16, 95% CI 0.03-0.30; no symptoms: *g*=0.21, 95% CI 0.11-0.030; *P*=.008). For depression and well-being, no significant differences were found between subgroups.

#### Meta-Regression Analysis

The meta-regression analysis (Table 2) revealed that study quality had a significant positive influence on the observed effects of anxiety but not on the remaining outcomes. Furthermore, a significant moderating impact of age on stress was observed, whereby online MBIs were found to be more effective in reducing stress in older samples. For mindfulness, the number of sessions had a significant negative influence on the effect size, with more sessions resulting in lower effect sizes.

**Table 2:** Meta-regression analyses^a^.

Outcome and predictor			*N*_comp_^b^		Slope		*Z*		*P* value
**Depression**									
	Study quality	88		0.05		1.09		.28	
	*N* sessions	84		0		0		.99	
	Mean age	80		0		0.47		.64	
	% female	87		0.01		1.37		.18	
**Anxiety**									
	Study quality	73		0.07		2.04		.04^c^	
	*N* sessions	69		0		–1.25		.21	
	Mean age	66		0		–0.03		.98	
	% female	73		0		0.83		.40	
**Stress**									
	Study quality	55		–0.04		–0.74		.46	
	*N* sessions	49		0.03		2.45		.05	
	Mean age	43		0.01		2.88		.004^d^	
	% female	55		0		0.25		.80	
**Well-being**									
	Study quality	51		0.07		0.90		.37	
	*N* sessions	49		0		–0.33		.74	
	Mean age	51		0		–0.28		.78	
	% female	51		0		0.17		.86	
**Mindfulness**									
	Study quality	71		0.01		0.26		.80	
	*N* sessions	66		–0.01		–2.80		.005^d^	
	Mean age	65		0		0.06		.95	
	% female	71		0.01		1.83		.07	

^a^Meta-regression analyses were conducted using a mixed effects model with unrestricted maximum likelihood.

^b^*N*_comp_: number of comparisons.

^c^*P*<.05.

^d^*P*<.01.

### Pre-to-Follow-up Between-Group Effects

#### Main Findings

The pre-to-follow-up effects are shown in Table 3. At follow-up, significant small effects were found for depression (26 comparisons: *g*=0.25, 95% CI 0.12-0.38, *P*<.001), and anxiety (21 comparisons: *g*=0.23, 95% CI 0.13-0.32; *P*<.001). The effects of stress were in favor of control conditions (15 comparisons: *g*=–0.24, 95% CI –0.40 to –0.09, *P*=.003). Effect sizes for well-being (18 comparisons: *g*=–0.02, 95% CI –0.53 to 0.49, *P*=.08) and mindfulness (27 comparisons: *g*=0.06, 95% CI –0.05 to 0.16, *P*=.28) were not significant. Heterogeneity varied considerably, from *I^2^*=36.49 for stress to *I^2^*=98.73 for well-being. After the removal of outliers, effect sizes for depression and mindfulness remained fairly the same (depression: *g*=0.27, 95% CI 0.14-0.40, *P*<.001; mindfulness: *g*=0.05, 95% CI –0.03 to 0.14, *P*=.21), with no changes in the significance of the effect and the level of heterogeneity remaining moderate (*I^2^*=40.36-53.930). For stress, the effect size was higher after removing outliers (stress: *g*=–0.11, 95% CI –0.21 to –0.02, *P*=.02). The fixed effects models showed similar results. For anxiety and well-being, no outliers were detected. When only medium- and high-quality studies were included, similar effect sizes were found for all outcomes, except for well-being which showed a substantial increase in effect size from *g*=–0.02 to *g*=0.17 (nonsignificant, 95% CI –0.03 to –0.37, *P*=.09). Effect sizes for depression, anxiety, and stress remained significant (depression: *g*=0.24, 95% CI 0.09-0.38, *P*=.001; anxiety: *g*=0.21, 95% CI 0.07-0.34, *P*=.003; stress: *g*=–0.30, 95% CI –0.51 to 0.08, *P*=.008), with moderate heterogeneity (depression: *I^2^*=53.21; anxiety: *I^2^*=43.56; stress: *I^2^*=71.02). The effect size for mindfulness remained nonsignificant (*g*=0.08, 95% CI –0.08 to 0.23; *P*=.34), with moderate heterogeneity (*I^2^*=70.06).

**Table 3:** Pre-to-follow-up effects of online mindfulness-based interventions compared with controls^a^.

Outcomes		*N*_comp_^b^	Hedges *g* (95% CI))	*Z*	Heterogeneity			Fail-safe *N*
					*Q* value	*I* ^2^		
**All studies (including outliers)**								
	Depression	26	0.25 (0.12 to 0.38)	3.80^c^	74.12^c^	66.27	201	
	Anxiety	21	0.23 (0.13 to 0.32)	4.62^c^	31.49^d^	36.49	189	
	Stress	15	–0.24 (–0.40 to –0.08)	–2.97^e^	37.09^e^	62.25	71	
	Well-being	18	–0.02 (–0.53 to 0.49)	–0.09	1254.86^c^	98.73	171	
	Mindfulness	27	0.06 (–0.05 to 0.16)	1.09	75.05^c^	65.36	0	
**All studies (excluding outliers)**								
	Depression^f^	25	0.27 (0.14 to 0.40)	4.18^c^	53.93^c^	55.50	220	
	Anxiety	N/A^g^	N/A	N/A	N/A	N/A	N/A	
	Stress^h^	13	–0.11 (–0.21 to –0.02)	–2.31^d^	9.35	0	12	
	Well-being	N/A	N/A	N/A	N/A	N/A	N/A	
	Mindfulness^i^	25	0.05 (–0.03 to 0.14)	1.23	40.36^d^	40.53	0	
**Medium- and high-quality studies**								
	Depression	16	0.24 (0.09 to 0.38)	3.23^e^	32.06^e^	53.21	75	
	Anxiety	13	0.21 (0.07 to 0.34)	2.99^e^	21.26^d^	43.56	45	
	Stress	11	–0.30 (–0.51 to –0.08)	–2.67^e^	34.51^c^	71.02	44	
	Well-being	13	0.17 (–0.03 to 0.37)	1.71	48.02^c^	75.00	24	
	Mindfulness	18	0.08 (–0.08 to 0.23)	0.96	56.78^c^	70.06	0	

^a^Only studies with a follow-up period of 1-3 months were included. Analyses were conducted using a random effects model.

^b^*N*_comp_: number of comparisons.

^c^*P*<.001.

^d^*P*<.05.

^e^*P*<.01.

^f^One outlier was removed: Mak et al [39].

^g^N/A: not applicable.

^h^Two outliers were removed: Kladnitski et al [34] (studies 1 and 3).

^i^Two outliers were removed: Pots et al [50] and Huberty et al [45].

#### Publication Bias

For depression, anxiety, and well-being, funnel plots were somewhat skewed in favor of studies with a positive outcome at follow-up. The trim-and-fill procedure by Duval and Tweedie indicated publication bias for all outcomes except stress; 1, 3, 6, and 6 studies were trimmed for depression, anxiety, well-being, and mindfulness, respectively. The adjusted effect sizes for depression (*g*=0.26, 95% CI 0.13-0.39), anxiety (*g*=0.18, 95% CI 0.07-0.29), and stress (*g*=–0.24, 95% CI –0.40 to –0.08) were similar to the unadjusted effect sizes, whereas effect sizes for mindfulness (*g*=-0.04, 95% CI –0.14 to 0.07) and well-being (*g*=-038, 95% CI –0.81 to 0.06) showed a considerable decline after adjusting for missing studies. Finally, the fail-safe *N* indicated that findings for depression, anxiety, and well-being may be considered robust, whereas this was not the case for stress and mindfulness. However, when only moderate-to-high quality studies were included in the analysis, for none of the remaining outcomes, the findings were deemed robust based on the fail-safe *N*.

## Discussion

### Principal Findings

An updated meta-analysis was conducted to assess the effects of the booming field of online MBIs on mental health across studies. In total, 97 RCTs were included in this meta-analysis, demonstrating the rapidly growing interest in implementing MBIs via eHealth platforms and apps. In comparison, only 15 RCTs were included in our meta-analysis conducted 5 years ago [28]. Overall, significant moderate pre-to-post effects were observed for depression (*g*=0.34), stress (*g*=0.44), and mindfulness (*g*=0.40), and a significant small effect was found for anxiety (*g*=0.26). After removing outliers and low-quality studies, similar results were found, except for well-being. Pre-to-follow-up analyses demonstrated significant small effects for depression and anxiety (*g*=0.25 and *g*=0.23, respectively). Our findings are largely in line with those reported in previous meta-analyses [9,10,28-30] and suggest that online MBIs have a significant low-to-moderate impact on mental health and that these effects are maintained at short-term follow-up.

Thus, when addressing depression and anxiety, the impact of online MBIs appears similar to MBIs in traditional face-to-face format [14,23], as well as to other common interventions such as cognitive behavioral therapy [134,135]. However, offering these interventions in an online format may have unique benefits of increased accessibility and scalability, thereby lowering the threshold for participation [136] and potential cost-effectiveness [137]. In addition, in the case of an app-based mode of delivery, the presence of a mobile device throughout most daily activities and experiences may facilitate the integration of newly learned skills into daily life [138,139]. Indeed, just-in-time information (eg, reminders) has been shown to contribute to the effectiveness of online MBIs [140]. Future research could further investigate the level of integration of skills learned in MBIs into daily life by using methods that allow for the assessment of daily fluctuations and situational contexts (eg, experience sampling methods) [141].

Our findings on well-being deserve special attention. In our updated meta-analysis, a significantly small effect on well-being was found, but only after omitting outliers (*g*=0.22) and low-quality studies (*g*=0.26). This is in contrast with our previous meta-analysis [28], where there was no difference in the effect on well-being when including or excluding low-quality trials. At first glance, one may consider the increase in the proportion of low-quality trials among those trials assessing well-being, from 13% (1/8 studies) to 36% (14/39 studies), as a possible explanation. As a result, studies of low quality may exert a larger influence on the effect size for well-being. However, this line of thought seems contradictory to our finding that study quality is not a moderator of the effect of online MBIs on well-being. Another potential explanation for this finding is that we used a different method to calculate the effect sizes for well-being. In our previous meta-analysis, we used the most valid outcome measure to compute the effect size. However, following the conceptualization of well-being by Keyes [142], in this meta-analysis, we computed combined effect sizes incorporating emotional, psychological, and social dimensions of well-being, where possible. Although we feel that this is the preferred method, this might have led to somewhat different results. In addition, we recognize a growing variability across studies as to how well-being is conceptualized and measured, which might have impacted the results. This is reflected in the increased levels of heterogeneity compared with our previous meta-analysis. Although heterogeneity was low to moderate (*I*^2^=32.86, when including all studies) in our meta-analysis published in 2016 [28], we found a heterogeneity level approaching 100% (*I*^2^=96.64) in this study. This calls for a more consistent assessment of well-being in future trials assessing the effectiveness of online MBIs. We support the recommendation by Chakhssi et al [143] that studies investigating the effects of interventions, in this case online MBIs, on well-being ideally include validated measures for emotional, psychological, and social dimensions of well-being.

The finding that studies of moderate-to-high quality indicate a positive impact of online MBIs on well-being is important because interventions such as MBSR, MBCT, and ACT emphasize well-being as an intervention outcome. Its relevance is further underlined by increasing evidence that mental well-being and mental illness are related yet discernible phenomena [144-146] and that higher levels of mental well-being reduce the incidence of mental health problems [147-149].

Remarkably, where online MBSR and online MBCT were the most prominent online MBIs 5 years ago, in this meta-analysis, online ACT dominates the field; 29 studies evaluated the impact of online ACT interventions compared with 5 studies in 2016 [28]. ACT is a distinct model of behavioral therapy, emphasizing the context and function of psychological phenomena as the target of psychological treatment [7]. Moderate-to-large effects on mental health have been found across studies evaluating the impact of ACT in clinical and nonclinical populations [21,150]. This meta-analysis thus shows that ACT is increasingly implemented as an online intervention in comparison with other types of MBIs. We found significant small effects of online ACT on depression (*g*=0.35), anxiety (*g*=0.23), and well-being (*g*=0.21), which is consistent with the findings of two other meta-analyses on the effectiveness of web-delivered ACT [151,152].

Whereas online ACT may have become an increasingly common type of intervention in this field of study, another development that is mirrored by our findings is that interventions are increasingly nonspecific. In 42.5% (53/125) of the comparisons, mixed or hybrid interventions, encompassing elements of both MBSR and MBCT as well as other mindfulness-based exercises, were used. We found that these programs prove to be effective in reducing symptoms of depression (*g*=0.37), anxiety (*g*=0.30), and stress (*g*=0.44), as well as in improving well-being (*g*=0.30) and mindfulness (*g*=0.40), an important finding considering that hybrid interventions are increasingly conquering the market of online MBIs. Interestingly, the type of intervention was not found to be a significant moderator of the effectiveness of online MBIs, suggesting that MBSR, MBCT, ACT, and hybrid MBIs are equally effective in improving mental health.

Subgroup analyses yielded significant differential effects of guidance, symptoms, and type of control group on stress, anxiety, and mindfulness. The effects of online MBIs on stress were significantly higher for interventions with therapist guidance (*g*=0.42) than for interventions without guidance (*g*=0.21). This is in line with our previous meta-analysis [28] and evidence demonstrating that guided interventions are more effective in reducing distress than unguided interventions [153]. Furthermore, the effects on anxiety were higher for samples with psychological symptoms than for those with physical or no symptoms. Populations without psychological symptoms may have less room to improve their psychological symptoms, due to lower baseline scores for anxiety (ie, a floor effect). In addition, the effects on stress and mindfulness were significantly larger when comparing online MBIs with inactive versus active control groups. In this regard, it should be noted that levels of heterogeneity were substantial, that is, it is questionable that indeed the types of subgroups are responsible for the differential outcomes. However, these results are in line with the common finding that effect sizes are related to the type of control group [154], with waiting list control groups typically yielding the largest effects [155].

Although the field of online mindfulness is booming, we noticed a number of undesirable phenomena that may undermine the accumulation of unbiased scientific knowledge in this specific domain, thereby hampering the development and optimization of novel online MBIs. The first phenomenon was related to adherence. Adherence is an important topic in the context of online interventions [156] and can be defined as the proportion of an intervention a person engages with or completes [157]. In this meta-analysis, it was found that although 70% (68/97) of studies reported relevant information regarding adherence to the intervention (eg, time spent on the intervention and number of modules started), only in 23% (22/97) of studies a definition or cut-off was provided for determining adherence versus nonadherence. This corroborates with an important systematic review demonstrating that a minority of studies evaluating eHealth interventions described a threshold for the intended use of the technology and that only 10% of the included studies reported a justification of the intended use [158]. The clinical relevance of online interventions is clear, as poor adherence may limit the effects of an intervention as a suboptimal dosage of the treatment may be received. For online MBIs, the relevance may be even bigger as regular practice of mindfulness is assumed to be essential for the development of mindfulness skills [159]. Therefore, describing a justified threshold for intended use that is aligned with the aim of the technology seems not only relevant but also important for future studies, as this is the basis for a more precise evaluation of adherence and the impact of an intervention.

The indications for publication bias that were found for all outcomes except stress represent a second phenomenon. The pre-post effect size for well-being was substantially reduced after adjusting for missing studies, whereas pre-post findings for depression and anxiety indicated the opposite. We encourage researchers and publishers to publish not only studies with positive outcomes but also studies with nonsignificant or negative findings to overcome the accumulation of unbiased scientific knowledge and the unduly hampering optimization of novel online MBIs.

A third phenomenon that should be addressed is an increase in the proportion of studies with a high risk of bias from 20% (3/15 studies) in our previous meta-analysis [28] to 34% (33/97 studies) in this meta-analysis. In addition, in our meta-analysis, low trial quality resulted in biased effect sizes for well-being. This is an undesirable trend that potentially undermines the reliability of research in this specific domain. Most studies did not meet two of the seven criteria used for rating the quality of studies. Nearly 78% (76/97) of the studies did not perform an adequate drop-out analysis, and 51% (49/97) of the studies did not conduct adequate power analysis. Therefore, attention to these specific analyses in future studies is highly recommended.

### Limitations

This meta-analysis included a large number of studies, which allowed moderator analyses and long-term follow-up measurements. However, some important limitations of this study must be considered. There was great variability in follow-up measurements, and the studies included in this meta-analysis only allowed for an overall assessment of effects until the 3-month follow-up. Owing to the limited number of studies using longer follow-up times (longer than 3 months), it remains unclear whether the effects of online MBIs remain at long-term follow-up. It should also be noted that heterogeneity was high for most moderator analyses. This suggests that other, yet unknown, factors may explain the effect differences rather than the observed factors.

### Conclusions

This updated meta-analysis not only demonstrates that the field of online MBIs is booming, with a significant low-to-moderate impact on mental health, but also corroborates previous evidence that online MBIs are beneficial for a wide range of populations and symptoms. Future trials assessing the effectiveness of online MBIs should focus on methodological quality parameters, on a priori definition and monitoring of adherence, and on longer follow-up measurements.

## Appendix

Multimedia Appendix 1 Study characteristics and outcome measures.

Multimedia Appendix 2 Methodological quality of studies included in the meta-analysis.

Multimedia Appendix 3 Subgroup analyses.

## Abbreviations

ACT: Acceptance and Commitment Therapy
MBCT: Mindfulness-Based Cognitive Therapy
MBI: mindfulness-based intervention
MBSR: Mindfulness-Based Stress Reduction
PRISMA: Preferred Reporting Items for Systematic Reviews and Meta-Analyses
RCT: randomized controlled trial
