# Supplementary material for: New Evidence in the Booming Field of Online Mindfulness: An Updated Meta-analysis of Randomized Controlled Trials
Source: JMIR Ment Health. 2021 Jul 19;8(7):e28168. doi: 10.2196/28168 (PMC8329762; doi:10.2196/28168)
Supplement: Multimedia Appendix 1 [file mental_v8i7e28168_app1.docx]

Multimedia Appendix 1a*.* Characteristics of studies included in the meta-analysis

| First author (year) | Population, country | % F^a^ | Mean age (range or *SD*)^b^ | Intervention (*n*) | Guidance (with/ without) | Delivery mode | *n* sessions, duration in weeks | Control group (*n*) | Measurements^c^ |
| --- | --- | --- | --- | --- | --- | --- | --- | --- | --- |
| Ahmad (2020) | University students, Canada | 75.2% | 24.8 (6.5) | MBI (35) | Without | Website | 12 sessions, 8 weeks | Waitlist (39) | Pre, mid, post (ITT) |
| Allexandre (2016) | Employees, US | 83.1% | 40.0 (12.8) | MBSR (33) | With (G) | Website | 8 sessions, 8 weeks | Waitlist (37) | Pre, post, 2-month FU (CA^d^) |
|  |  |  |  | MBSR (54) | Without | Website | 8 sessions, 8 weeks | Waitlist (37) | Pre, post, 2-month FU (CA^d^) |
| Aikens (2014)* | Employees, US | 50.0 | U (U) | MBSR (44) | With (G + I) | Website and virtual online classroom | 7 sessions, 7 weeks | Waitlist (45) | Pre, post (ITT) |
| Barrett (2020) | Social and healthcare workers, Ireland | 88% | 37.12 (12.18) | ACT (22) | Without | Website | 3 sessions, 2 weeks | CBT (20) | Pre, post (ITT) |
| Beshai (2020) | Distressed adults, US | 43.9% | 35.13 (10.57) | MBI (227) | Without | Website | 4 sessions, 4 weeks | Nature images (229) | Pre, post (ITT) |
| Boettcher (2014)* | Individuals with anxiety disorder, Sweden | 71.4 | 38 (10.3, 22-65) | MBI (45) | Without | Website | 8 sessions, 8 weeks | Online discussion forum (46) | Pre, post (ITT) |
| Bostock (2018) | Employees, UK | 59.2 | 35.5 (7.7, 23-61) | MBI (128) | Without | App | 45 sessions, 8 weeks | Waitlist (110) | Pre, post (CA) |
| Bruggeman-Everts (2017) | Severely fatigued cancer patients, the Netherlands | 73.7 | 54.8 (10.3) | MBCT (55) | With (I) | Website | 9 sessions, 9 weeks | Psycho-education (50) | Pre, post (ITT) |
|  |  |  |  |  |  |  |  | AAF (62) | Pre, post (ITT) |
| Buhrman (2013)* | Chronic pain patients, Sweden | 59.2 | 49.1 (10.3, 27-69) | ACT (38) | With (I) | Website | 7 sessions, 7 weeks | Online discussion forum (38) | Pre, post (ITT) |
| Cavalera (2018) | Multiple Sclerosis patients, Italy | 64.5 | 42.8 (8.7) | MBSR (69) | With (G) | Website | 8 sessions, 8 weeks | Online psycho-education (70) | Pre, post, 6-month FU (ITT) |
| Cavanagh (2013)* | Students, UK | 88.5 | 24.7 (6.4, 19-51) | MBI (54) | Without | Website | U, 2 weeks | Waitlist (50) | Pre, post (ITT) |
| Cavanagh (2018) | University students and staff, UK | 80.0 | 31.0 (18-68) | MBI (53) | Without | Website | 14 sessions,  2 weeks | Waitlist (50) | Pre, post (ITT) |
|  |  |  |  |  |  |  |  | Online psycho-education (52) | Pre, post (ITT) |
| Champion  (2018) | Employees, US | 59.7^e^ | 39.4 (5.8, 25-59)^e^ | MBI (38) | Without | App | 30 sessions,  4 weeks | Waitlist (36) | Pre, post (ITT) |
| Chapoutot (2020) | Adults with chronic insomnia or hypnotic dependence, France | 80% | 48 (10) | ACT (15) | With (I) | Videoconferencing | 4 sessions, 8 weeks | Waitlist (15) | Pre, post, 6-month FU (CA) |
| Compen (2018) | Distressed cancer patients, the Netherlands | 85.1 | 51.5 (10.4) | MBCT (90) | With (I) | Website | 8 sessions, 8 weeks | TAU (78) | Pre, post (ITT) |
| Cox (2018) | Intensive care unit patients treated for cardiorespiratory failure, US | 40.8 | 50.4 (14.4) | MBSR (31) | Without | App | 4 sessions, 4 weeks | Online psycho-education (18) | Pre, post, 2-month FU (CA) |
| De Wit (2020) | Partners of patients with ALS or PMA, The Netherlands | 64.9% | 62 (10,14) | ACT (74) | With (I) | Website | 7 sessions, 12 weeks | Waitlist (74) | Pre, post, 6-month FU (ITT) |
| Dowd (2015)* | Individuals with self-reported chronic pain, Ireland | 90.3 | 44.5 (12.3, 19-76) | MBCT (62) | Without | Website | 12 sessions, 6 weeks | Psycho-education (62) | Pre, post, 6-month FU (ITT) |
| El Morr (2020) | Students, Canada | 78.6% | 22.55 (6.1) | MBI (80) | Without | Website | 12 sessions, 8 weeks | Waitlist (80) | Pre, post (ITT) |
| Eriksson (2018) | Practising psychologists, Sweden | 96.0 | 36.2 (8.2, 24-57) | CBI (52) | Without | Website | 6 sessions, 6 weeks | Waitlist (49) | Pre, post (CA^d^) |
| Eustis (2018) | Students, US | 78.8 | 25.4 (7.9) | ABI (78) | With (I) | Website | 3 sessions, 4 weeks | Waitlist (78) | Pre, post, 1-month FU (CA) |
| Flett (2018) | Students, New Zealand | 70.2 | 20.1 (2.9, 18-49) | MBI (72) | Without | App (Headspace) | 10 sessions, 10 days | Evernote app (75) | Pre, post, 1-month FU (CA) |
|  |  |  |  | MBI (63) | Without | App (Smiling Mind) | 10 sessions, 10 days | Evernote app (75) | Pre, post, 1-month FU (CA) |
| Forbes (2020) | Women with chronic pelvic pain, UK | 100% | U (U) | MBI (31) | Without | App | 60 sessions, 8.5 weeks | Muscle relaxation (30) | Pre, post, 1-month FU, 4-month FU (ITT) |
|  |  |  |  | MBI (31) | Without | App | 60 sessions, 8.5 weeks | TAU (29) | Pre, post, 1-month FU, 4-month FU (ITT) |
| Gaigg (2020) | Adults with autism, UK | 82,1% | 41.71 (11.01) | MBCT (19) | Without | Website | 10 sessions, 8 weeks | CBT (16) | Pre, post, 2.5-month and 5-month FU (CA) |
|  |  |  | 44.09 (11.93) | MBCT (19) | Without | Website | 10 sessions, 8 weeks | WLC (19) | Pre, post, 2.5-month and 5-month FU (CA) |
| Gao (2021) | Adults with sleep-interfering worry, US | 80,3% | 41 (18) | MBI (36) | Without | App | 30 sessions, 8 weeks | TAU (35) | Pre, mid, post, 3-month FU (ITT) |
| Garrison (2018) | Smokers seeking to quit, US | 71.7 | U (U) | MBI + online ES (245) | Without | App | 22 sessions, 22 days | Online ES (260) | Pre, post (CA) |
| Gili (2020) | Mild and moderate depressed patients, Spain | 77.8 | 45.3 (12.6) | MBI + TAU (54) | Without | Website | 4 sessions, 8 weeks | TAU (57) | Pre, post, 6-month FU, 12-month FU (ITT) |
|  |  |  |  |  |  |  |  | TAU + online psycho-education (54) | Pre, post, 6-month FU, 12-month FU (ITT) |
|  |  |  |  |  |  |  |  | TAU + online positive affect intervention (56) | Pre, post, 6-month FU, 12-month FU (ITT) |
| Glück (2011)* | Students and employees, Austria/ Switzerland | 73.5 | 35.2 (20-73) | MBSR (28) | Without | Website | 2 sessions, 2 weeks | Waitlist (21) | Pre, post (ITT) |
| Gu (2018) | University students and staff, UK | 72.9 | 24.2 (18-49) | MBI (83) | Without | Website | U, 2 weeks | Waitlist (63) | Pre, post (CA) |
|  |  |  |  |  |  |  |  | Online classical music (68) | Pre, post (CA) |
| Hearn (2018) | Spinal cord injury patients, UK | 54.0 | 44.4 (10.4) | MBI (36) | Without | Website | 8 sessions, 8 weeks | Psycho-education (31) | Pre, post, 3-month FU (CA) |
| Hearn (2019) | Family caregivers of people with spinal cord injuries or chronic neuropathic pain, UK | 47.3 | 44.0 (11.1) | MBI (28) | Without | Website | 8 sessions, 8 weeks | Psycho-education (27) | Pre, post, 3-month FU (CA) |
| Henriksson (2016) | Chronic pain patients, Sweden | 93.5 | 51.0 (9.3) | MBSR (55) | Without | Website | 8 sessions, 8 weeks | Online discussion forum (52) | Pre, post (CA) |
| Hesser (2012)* | Tinnitus patients, Sweden | 43.4 | 48.5 (14.7, 20-78) | ACT (35) | With (I) | Website | 8 sessions, 8 weeks | Online discussion forum (32) | Pre, post (CA^d^) |
|  |  |  |  |  |  |  |  | Online CBT (32) | Pre, post, 10-month FU (CA^d^) |
| Hoffmann (2020) | Adults with severe health anxiety, Denmark | 65,3% | 39.59 (9.93) | ACT (53) | With (I) | Web app | 7 sessions, 12 weeks | Discussion forum (48) | Pre, mid, post, 6-month FU (ITT) |
| Howells (2016) | General population, Australia, US, Poland, UK, Malta, Ireland, Switzerland, Sweden, New Zealand, Singapore | 88.1 | 40.7 (10.6) | MBI (97) | Without | App | 10 sessions, 10 days | Online neutral task (97) | Pre, post (CA) |
| Huberty (2019) | Students, US | 89.8^d^ | 21.2 (4.9) | MBI (56) | Without | App | 7 sessions, 8 weeks | Waitlist (53) | Pre, post, 1-month FU (CA) |
| Ivtzan (2018) | General population, UK | 80.7 | 39.8 (15.4) | MBI (33) | Without | Website | 8 sessions, 8 weeks | Waitlist (29) | Pre, post (ITT) |
|  | General population, China | 58.5 | 24.4 (8.6) | MBI (27) | Without | Website | 8 sessions, 8 weeks | Waitlist (26) | Pre, post (ITT) |
| Jelinek (2020) | Adults with depressive symptoms, Germany | 82.6% | 45.41 (10.55) | MBCT (32) | Without | Website | 14 sessions, 2 weeks | Behavioural activiation (37) | Pre, post, 1-month FU (ITT) |
|  |  |  | 46.42 (10.28) | MBSR (32) | Without | Website | 14 sessions, 2 weeks | TAU (35) | Pre, post, 1-month FU (ITT) |
| Khazaeili (2019) | Caregivers of women with MS, Iran | U | U (U) | MBI (15) | With (U) | App | 8 sessions, 8 weeks | No intervention (15) | Pre, post, 1-month FU (U) |
| Kladnitski (2020) | Adults with an anxiety or depressive disorder, Australia | 84,8% | U (U) | MBI (40) | With (I) | Website | 6 sessions, 14 weeks | TAU (39) | Pre, mid, post, 3-month FU (ITT) |
|  |  |  |  | MBI (40) | With (I) | Website | 6 sessions, 14 weeks | CBT (39) | Pre, mid, post, 3-month FU (ITT) |
|  |  | U | U (U) | MBCT (40) | With (I) | Website |  | TAU (39) | Pre, mid, post, 3-month FU (ITT) |
|  |  |  |  | MBI (40) | With (I) | Website | 6 sessions, 14 weeks | CBT (39) |  |
| Krieger (2019) | Individuals with high self-criticism, Switzerland | 77.7 | 37.7 (11.5) | MBCL (59) | With (I)^f^ | Website | 7 sessions, 8 weeks | Waitlist (62) | Pre, post (ITT) |
| Köhle (2021) | Partners of cancer patients, the Netherlands | 70.4 | 55.9 (10.7, 27-82) | ACT + self-compassion (67) | With (I) | Website | 8 sessions, 12 weeks | Waitlist (66) | Pre, post (ITT) |
|  |  |  |  | ACT + self-compassion (70) | Without^g^ | Website | 8 sessions, 12 weeks | Waitlist (66) | Pre, post (ITT) |
| Krusche (2018) | Pregnant women, UK | 100 | 32.7 (22-40)^e^ | MBI (107) | Without | Website | 10 sessions, 4 weeks | Waitlist (78) | Pre, post (CA) |
| Kubo (2019) | Cancer patients, US | 68.0 | 58.1 (14.4) | MBI (54) | Without | App | U, 8 weeks | Waitlist (43) | Pre, post (CA) |
|  | Caregivers of cancer patients, US | 58.1 | 57.6 (17.7) | MBI (17) | Without | App | U, 8 weeks | Waitlist (14) | Pre, post (CA) |
| Kubo (2020) | Adults with cancer, US | 69,9% | 66.5 (9.6) | MBI (52) | Without | App | 42 sessions, 6 weeks | WLC (51) | Pre, post, 1.5-month FU (CA) |
|  | Informal caregivers of adults with cancer, US | 79% | 62.4 (13.4) | MBI (22) |  |  |  | WLC (17) |  |
| Kvillemo (2016) | Students, Sweden | 57.9 | 26.2 (5.8) | MBSR (40) | Without | Website | 8 sessions, 8 weeks | Online expressive writing (36) | Pre, post (ITT) |
| Lappalainen (2015) | Individuals depressive symptoms, Finland | 71.8 | 51.9 (12.9) | ACT (19) | With (I) | Website | 6 sessions, 7 weeks | Waitlist (20) | Pre, post (ITT) |
| Lee (2018) | Healthcare providers, Korea | 100 | 35.4 (7.2)^e^ | MBI (19) | Without | Website | 20 sessions, 4 weeks | Waitlist (18) | Pre, post (CA) |
| Levin (2014)* | Students, US | 53.9 | 18.4 (0.5, 18-20) | ACT (37) | Without | Website | 2 sessions, 3 weeks | Waitlist (39) | Pre, post (ITT) |
| Levin (2016) | Undergraduate university students, US | 76.9 | 21.6 (5.5, 18-58) | ACT (110) | Without | Website | 2 sessions, 3 weeks | Online psycho-education (118) | Pre, post, 1-month and 3-month FU (ITT) |
| Levin (2017) | College students, US | 66.0 | 20.5 (2.7) | ACT (40) | Without | Website | 6 sessions, 4 weeks | Waitlist (39) | Pre, post (ITT) |
| Levin (2019) | Community sample, US | 68.1 | 21.9 (5.5, 18-46) | ACT + online experience sampling (23) | Without | App – tailored modules | 4 sessions, 4 weeks | Online experience sampling (24) | Pre, post (ITT) |
|  |  |  |  | ACT + online experience sampling (22) | Without | App – random modules | 4 sessions, 4 weeks | Online experience sampling (24) | Pre, post (ITT) |
| Levin (2020) | University students, US | 100% | 20.43 (2.47) | MBI (10) | Without | App | 28 sessions, 4 weeks | WLC (13) | Pre, mid, post (CA) |
| Levin (2020) | University students, US | 72.4% | 22.27 (5.08) | ACT (45) | With (I) | Website | 12 sessions, 6 weeks | WLC (45) | Pre, post, 2-month FU (ITT) |
| Lilly (2019) | Emergency medical dispatchers, US and Canada | 81.9 | U (U) | MBSR (163) | Without | Website | 7 sessions, 7 weeks | Waitlist (160) | Pre, post, 3-month FU (ITT^h^) |
| Lin (2017) | Individuals with self-reported chronic pain, Germany | 84.1 | 51.7 (13.1) | ACT (100) | With (I) | Website | 7 sessions, 7 weeks | Waitlist (101) | Pre, post, 4-month FU (ITT) |
|  |  |  |  | ACT (101) | Without | Website | 7 sessions, 7 weeks | Waitlist (101) | Pre, post, 4-month FU (ITT) |
| Ly (2014)* | MDD patients, Sweden | 70.4 | 36.0 (10.8, 20-61) | MBCT (41) | With (I) | App | U, 8 weeks | BA treatment (40) | Pre, post, 4-month FU (ITT) |
| Ma (2018) | Community sample, China | 57.9 | 27.8 (7.9, 18-47) | MBCT + online group discussion sessions (48) | Without | Website | 8 sessions, 8 weeks | Online discussion forum (48) | Pre, post (CA) |
|  |  |  |  |  |  |  |  | No intervention (48) | Pre, post (CA) |
|  |  |  |  | MBCT (48) | Without | Website | 8 sessions, 8 weeks | Online discussion forum (48) | Pre, post (CA) |
|  |  |  |  |  |  |  |  | No intervention (48) | Pre, post (CA) |
| Mak (2015)* | Students and employees, China | 66.3 | 22.8 (6.5, 17-53) | MBSR (107) | Without | Website | 8 sessions, 8 weeks | Waitlist (107) | Pre, post, 1-month FU (ITT) |
|  |  |  |  | MBSR-HAPA (107) | Without | Website | 8 sessions, 8 weeks | Waitlist (107) | Pre, post, 1-month FU (ITT) |
| Mak (2017) | Students and employees, China | 74.3 | 32.6 (12.5) | MBSR (604) | Without | Website | 8 sessions, 8 weeks | Online CBT (651) | Pre, post, 3-month FU (ITT) |
| Mak (2018) | General population, China | 72.9 | 33.6 (12.1, 18-83) | MBSR (703) | Without | App and website | 28 sessions, 4 weeks | Online cognitive behavioural psycho-education (753) | Pre, post, 3-month FU (ITT) |
|  |  |  |  | CBI (705) | Without | App and website | 28 sessions, 4 weeks | Online cognitive behavioural psycho-education (753) | Pre, post, 3-month FU (ITT) |
| Messer (2019) | Cancer survivors, US | 70% | 51.0 (10.58) | MBSR (11) | Without | Website | 6 weeks, 6 sessions | TAU (10) | Pre, post (ITT) |
| Milbury (2020) | Adults with brain cancer, US | 46% | 57.49 ( 31-75) | MBI (18) | Without | Videoconferencing | 4 weeks, 4 sessions | TAU (17) | Pre, post, 2-month FU (ITT) |
| Molander (2018) | Individuals with hearing loss, Sweden | 67.2 | 58.7 (12.8, 25-83) | ACT (31) | With (I) | Website | 8 sessions, 8 weeks | Waitlist (30) | Pre, post (ITT) |
| Morledge (2013)* | Community sample, US | 88.9 | U | MBSR (184) | With (G) | Website | 8 sessions, 8 weeks | Waitlist (184) | Pre, post, 1-month FU (ITT) |
|  |  |  |  | MBSR (183) | Without | Website | 8 sessions, 8 weeks | Waitlist (184) | Pre, post, 1-month FU (ITT) |
| Nadler (2020) | Adult employees, US | 73.5% | u (range 18-60+) | MBI (37) | Without | Website | 8 weeks, 8 sessions | WLC (65) | Pre, post (CA) |
| Nguyen-Feng (2016) | Students, US | 66.5 | U | MBI + Present control (102) | Without | Website | 3 sessions, 4 weeks | Online present control (99) | Pre, post (ITT) |
| Nguyen-Feng (2017) | Psychology students, US | 66.0 | U | MBI (122) | Without | Website | 6 sessions, 4 weeks | Online psycho-education (122) | Pre, post, 1-month FU (CA) |
| Nissen (2020) | Cancer survivors with depressive or anxious symptoms, Denmark | 91.33% | 55.45 (9.94) | MBI (104) | Without | Website | 8 weeks, 8 sessions | WLC (46) | Pre, mid, post, 6-month FU (ITT) |
| Noone (2018) | Students, Ireland | 75.8 | 20.9 (4.4) | MBI (43) | Without | App | 30 sessions, 6 weeks | Online guided breathing exercises (48) | Pre, post (ITT) |
| O'Driscoll (2019) | Pharmacy students, Ireland | 76.9% | U (U) | MBI (19) | Without | Website | 4 weeks, 4 sessions | WLC (33) | Pre, post (CA) |
| Potharst (2019) | Mothers with elevated parental stress, The Netherlands | 100% | 36.22 (3.86) | MBI (37) | Without | Website | 8 weeks, 8 sessions | WLC (30) | Pre, post, 2.5-month FU (ITT) |
| Pots (2016)* | Individuals with mild to moderate depressive symptoms, the Netherlands | 75.8 | 46.9 (12.1, 20-73) | ACT (82) | With (I) | Website | 9 sessions, 12 weeks | Waitlist (87) | Pre, post, 3-month FU (ITT) |
|  |  |  |  |  |  |  |  | Online expressive writing (67) | Pre, post, 3-month FU (ITT) |
| Puzia (2020) | Adults with myeloproliferative neoplasm, US | 76.3% | U (U) | MBI (28) | Without | App | 28 sessions, 4 weeks | Educational information (51) | Pre, post (CA) |
| Querstret (2017, 2018) | Employees, UK | 80.5 | 40.7 (10.5, 21-62) | MBI (60) | Without | Website | 10 sessions, 4 weeks | Waitlist (58) | Pre, post, (ITT) |
| Räsänen (2016) | Students, Finland | 85.3 | 24.3 (3.3) | ACT (33) | With (I) | Website | 7 sessions, 7 weeks | Waitlist (35) | Pre, post, 12-month FU (ITT) |
| Ritvo (2020) | University students, Canada | 76% | 23.10 (8.09) | MBI (76) | Without | Website | 12 sessions, 8 weeks | WLC (78) | Pre, post (ITT) |
| Rosen (2018) | Breast cancer patients, US | 100 | 52.3 (10.3, 29-73) | MBI (57) | Without | App | 10 sessions, 8 weeks | Waitlist (55) | Pre, post, 1-month FU (CA^d^) |
| Russell (2018) | Melanoma patients, Australia | 53.6 | 53.4 (13.1) | MBI (46) | Without | Website | 6 sessions, 6 weeks | TAU (23) | Pre, post (CA) |
| Sagon (2018) | Students, US | 71.8 | 18.2 (0.4) | ABI (52) | Without | Website | U, 8 weeks | Waitlist (51) | Pre, post (ITT) |
| Sairanen (2019) | Parents of children with type 1 diabetes with burnout symptoms, Sweden | 81,1% | 42.62 (6.96) | ACT (37) | With (I) | Website | 5 sessions, 10 weeks | WLC (37) | Pre, post 4-month FU (CA) |
| Scott (2018) | Chronic pain patients, UK | 63.5 | 45.5 (14.0) | TAU + ACT (31) | With (I) | Website | 8 sessions, 12 weeks | TAU (32) | Pre, post, 6-month FU (ITT) |
| Segal (2020) | Adults with residual depressive symptoms, US | 75.6% | 48.3 (14.9) | MBCT (230) | With (I) | Website | 8 sessions, 12 weeks | TAU (230) | Pre, post, 12-month FU (ITT) |
| Shore (2018) | Community sample, UK | 89.0 | 32.2 (13.6, 18-67) | MBI (56) | Without | Website | 6 sessions, 2 weeks | Waitlist (54) | Pre, post (CA) |
| Simister (2018) | Fibromyalgia patients, Canada | 95.0 | 39.7 (9.4, 18-64) | TAU + ACT (33) | Without | Website | 7 sessions, 8 weeks | TAU (34) | Pre, post, 3-month FU (ITT) |
| Stjernswärd (2017) | Caregivers of patients with a mental illness, Sweden | 88.1 | 54.0 (40-69) | MBSR (78) | Without | Website | 8 sessions, 10 weeks | Waitlist (73) | Pre, post (CA) |
| Tighe (2017) | Indigenous people, Australia | 63.9 | 26.3 (8.1, 18-56) | ACT (31) | Without | App | 3 sessions, 6 weeks | Waitlist (30) | Pre, post (ITT) |
| Trompetter (2014)* | Individuals with self reported chronic pain, the Netherlands | 76.0 | 52.8 (20-84) | ACT (82) | With (I) | Website | 9 sessions, 12 weeks | Waitlist (77) | Pre, post, 3-month FU (ITT) |
|  |  |  |  |  |  |  |  | Online expressive writing (79) | Pre, post, 3-month FU (ITT) |
| Van Emmerik (2018) | Community sample, the Netherlands | 96.0 | 44.7 (9.8, 19-73) | MBSR (191) | Without | App | 5 sessions, 5 weeks | Waitlist (186) | Pre, post (ITT) |
| Viskovich (2019) | University students, Australia | 63.9% | 26.85 (8.77) | ACT (596) | Without | Website | 4 sessions, 4 weeks | WLC (566) | Pre, post. 2.5-month FU (ITT) |
| Wahbeh (2016) | Older adults, US | 50.0 | 76.2 (7.4) | MBI (8) | With (I) | Website | 6 sessions, 6 weeks | Online health education (8) | Pre, post (CA) |
| Wahbeh (2018) | Older adults with depressive symptoms, US | 80.0 | 64.8 (6.2) | MBI (26) | With (I) | Website | 6 sessions, 6 weeks | Waitlist (24) | Pre, post (CA) |
| Walsh (2019) | Students, Canada | 83.7 | 20.0 (2.5) | MBI (58) | Without | App | U, 3 weeks | Cognitive training app (50) | Pre, post (CA) |
| Wolever (2012)* | Employees, US | 77.2 | 43.0 (U) | MBSR (52) | With (G) | Virtual online classroom | 12 sessions, 12 weeks | No intervention (53) | Pre, post (ITT) |
|  |  |  |  |  |  |  |  | Yoga (90)^i^ | Pre, post (ITT) |
| Yang (2018) | Medical students, US | 63.6 | 25.1 (21-47) | MBI (45) | Without | App | 30 sessions, 4 weeks | Waitlist (43) | Pre, post, 1-month FU (CA) |
| Yang (2019) | Pregnant women depressive or anxiety symptoms, China | 100 | 30.8 (4.5) | MBSR (62) | With (I) | App | 4 sessions, 8 weeks | TAU (61) | Pre, post (ITT) |
| Zernicke (2014)* | Cancer recovery patients, Canada | 72.6 | 58.0 (29-79) | MBSR (30) | With (G) | Virtual online classroom | 8 sessions, 8 weeks | Waitlist (32) | Pre, post (ITT) |

*Note*. AAF, Ambulant Activity Feedback; ABI, Acceptance-Based Intervention; ACT, Acceptance and Commitment Therapy; BA, Behavioural Activation; CBT, Cognitive Behavioural Therapy; CBI, Compassion-Based Intervention; ES, experience sampling; F, female; FU, follow-up; G, group-based; HAPA, Health Action Process Approach; I, individual; ITT, intention-to-treat; MBCL, Mindfulness-Based Compassionate Living; MBCT, Mindfulness-Based Cognitive Therapy; MBI, Mindfulness-Based Intervention; MBSR, Mindfulness-Based Stress Reduction; MDD, Major Depressive Disorder; SD, standard deviation; TAU, treatment as usual; U, unknown; UK, United Kingdom; US, United States.

^a^% female of the total study population at baseline.

^b^Mean age (*SD* and/or range) of the total study population at baseline. If Means and SDs are reported for experimental and control conditions separately, weighted means and SDs are calculated by the authors.

^c^We solely report measurements that were used in the meta-analysis. Follow-up times are since post-intervention.

^d^Completer data distracted from paper is used in meta-analysis, actual analyses in paper are ITT.

^e^Based on completers data only.

^f^Guidance was only provided upon request.

^g^This condition received the intervention with automated feedback, but no therapist support was offered.
^h^ITT data on all participants that completed at least one survey (excluding 2 participants after randomisation).

^i^This comparison is not included in our analyses due to lack of data.

*Studies included in the meta-analysis of Spijkerman et al. (2016).

Multimedia Appendix 1b*.* Outcome measures used in studies included in the meta-analysis

| First author (year) | Outcome measures^a^ | | | | |
| --- | --- | --- | --- | --- | --- |
|  | Depression | Anxiety | Stress | Well-being | Mindfulness |
| Ahmad (2020) | PHQ-9 | BAI | PSS | - | FFMQ-SF |
| Allexandre (2016) | - | - | PSS | SF-36-EW | MAAS |
|  | - | - | PSS | SF-36-EW | MAAS |
| Aikens (2014)* | - | - | PSS | - | FFMQ |
| Barrett (2020) | - | - | PSS | - | - |
| Beshai (2020) | PHQ-9 | GAD-7 | PSS | - | FFMQ-15 |
| Boettcher (2014)* | BDI-II | BAI | - | QOLI | - |
| Bostock (2018) | HADS-D | HADS-A | - | WEMWBS | - |
| Bruggeman-Everts (2017) | - | - | - | PANAS-PA | - |
|  | - | - | - | PANAS-PA | - |
| Buhrman (2013)* | HADS-D | HADS-A | - | QOLI | - |
| Cavalera (2018) | HADS-D | HADS-A | - | - | - |
| Cavanagh (2013)* |  | - | PSS | - | FFMQ |
| Cavanagh (2018) |  | - | PSS | - | FFMQ |
|  |  | - | PSS | - | FFMQ |
| Champion  (2018) | - | - | PSS | SWLS | - |
| Chapoutot (2020) | - | - | - | - | MAAS |
| Compen (2018) | HADS-D | HADS-A | - | MHC-SF | FFMQ-SF |
| Cox (2018) | PHQ-9 | GAD-7 | - | - | CAMS-R |
| De Wit (2020) | HADS-D | HADS-A | - | - | - |
| Dowd (2015)* | HADS-D | HADS-A | - | SWLS | MAAS |
| El Morr (2020) | PHQ-9 | BAI | PSS | - | FFMQ-SF |
| Eriksson (2018) | - | - | PSS | - | FFMQ |
| Eustis (2018) | DASS-D | DASS-A | DASS-S | QOLI | - |
| Flett (2018) | CES-D | HADS-A | PSS | FS | CAMS-R |
|  | CES-D | HADS-A | PSS | FS | CAMS-R |
| Forbes (2020) | HADS-D | HADS-A | - | - | CAMS-R |
|  | HADS-D | HADS-A | - | - | CAMS-R |
| Gaigg (2020) | HADS-D | GAD-7 | - | - | FFMQ-NR |
|  | HADS-D | GAD-7 | - | - | FFMQ-NR |
| Gao (2021) | - | GAD-7 | - | - | FFMQ |
| Garrison (2018) | - | - | - | - | FFMQ |
| Gili (2020) | PHQ-9 | - | - | PANAS-PA | - |
|  | PHQ-9 | - | - | PANAS-PA | - |
|  | PHQ-9 | - | - | PANAS-PA | - |
| Glück (2011)* | - | - | PSQ | - | FMI |
| Gu (2018) | - | - | PSS | - | FFMQ |
|  | - | - | PSS | - | FFMQ |
| Hearn (2018) | HADS-D | HADS-A | - | - | FFMQ |
| Hearn (2019) | HADS-D | HADS-A | - | - | FFMQ |
| Henriksson (2016) | - | - | - | LiSat-11 | FFMQ |
| Hesser (2012)* | HADS-D | HADS-A | PSS | QOLI | - |
|  | HADS-D | HADS-A | PSS | QOLI | - |
| Hoffmann (2020) | SCL92-D | SCL92-A | - | - | - |
| Howells (2016) | CES-D | - | - | FS, PANAS-PA, SWLS | - |
| Huberty (2019) | - | - | PSS | - | FFMQ |
| Ivtzan (2018) | - | - | - | PANAS-PA, MLQ-P | FMI-SF |
|  | - | - | - | PANAS-PA, MLQ-P | FMI-SF |
| Jelinek (2020) | PHQ-9 | - | - | - | KIMS |
|  | PHQ-9 | - | - | - | KIMS |
| Khazaeili (2019) | BDI-II | BAI-II | - | - | FFMQ |
| Kladnitski (2020) | PHQ-9 | GAD-7 | K-10 | - | - |
|  | PHQ-9 | GAD-7 | K-10 | - | - |
|  | PHQ-9 | GAD-7 | K-10 | - | - |
|  |  |  |  |  |  |
| Krieger (2019) | DASS-D | DASS-A | DASS-S | SWLS | CHIME |
| Köhle (2021) | HADS-D | HADS-A | - | MHC-SF | - |
|  | HADS-D | HADS-A | - | MHC-SF | - |
| Krusche (2018) | EPDS | GAD-7 | PSS | - | FFMQ |
| Kubo (2019) | HADS-D | HADS-A | - | FACT-G | FFMQ-SF |
|  | HADS-D | HADS-A | - | - | FFMQ-SF |
| Kubo (2020) | HADS-D | HADS-A | NCCNDT | - | FFMQ‐SF |
|  |  |  |  |  |  |
| Kvillemo (2016) | CES-D | - | - | PWBS | - |
| Lappalainen (2015) | BDI-II | - | - | - | FFMQ |
| Lee (2018) | - | - | - | PANAS-PA | - |
| Levin (2014)* | DASS-D | DASS-A | DASS-S | - | - |
| Levin (2016) | DASS-D | DASS-A | DASS-S | MHC-SF | FFMQ |
| Levin (2017) | CCAPS-34-D | CCAPS-34-A | - | MHC-SF | - |
| Levin (2019) | DASS-D | DASS-A | DASS-S | MHC-SF | - |
|  | DASS-D | DASS-A | DASS-S | MHC-SF | - |
| Levin (2020) | CCAPS-D | CCAPS-A | CCAPS-S | MHC-SF | FFMQ |
| Levin (2020) | CCAPS-D | CCAPS-A | CCAPS-S | MHC-SF | - |
| Lilly (2019) | - | - | C-SOSI | - | MAAS |
| Lin (2017) | PHQ-9 | GAD-7 | - | - | - |
|  | PHQ-9 | GAD-7 | - | - | - |
| Ly (2014)* | BDI-II | BAI | - | QOLI | - |
| Ma (2018) | SDS | SAS | - | - | FFMQ |
|  | SDS | SAS | - | - | FFMQ |
|  | SDS | SAS | - | - | FFMQ |
|  | SDS | SAS | - | - | FFMQ |
| Mak (2015)* | DASS-D | DASS-A | PSS | SWLS, WBI | FFMQ |
|  | DASS-D | DASS-A | PSS | SWLS, WBI | FFMQ |
| Mak (2017) | MHI-D | MHI-A | - | SWLS, WBI | - |
| Mak (2018) | - | - | - | WBI | MAAS |
|  | - | - | - | WBI | MAAS |
| Messer (2019) | HADS-D | HADS-A | - | - | - |
| Milbury (2020) | CES-D | - | - | - | MAAS |
| Molander (2018) | PHQ-9 | GAD-7 | - | QOLI | - |
| Morledge (2013)* | - | - | PSS | - | MAAS |
|  | - | - | PSS | - | MAAS |
| Nadler (2020) | - | - | PSS | - | FFMQ-SF |
| Nguyen-Feng (2016) | DASS-D | DASS-A | PSS | - | - |
| Nguyen-Feng (2017) | DASS-D | DASS-A | PSS | - | - |
| Nissen (2020) | BDI-II | STAI-Y | PSS | WHO-5 | - |
| Noone (2018) | - | - | - | WEMWBS, PANAS-PA | FFMQ |
| O'Driscoll (2019) | - | - | PSS | - | FFMQ |
| Potharst (2019) | PHQ-4-D | PHQ-4-A | - | - | - |
| Pots (2016)* | CES-D | HADS-A | - | MHC-SF | FFMQ-SF |
|  | CES-D | HADS-A | - | MHC-SF | FFMQ-SF |
| Puzia (2020) | PROMIS-D | PROMIS-A | - | - | - |
| Querstret (2017, 2018) | PHQ-9 | GAD-7 | PSS | - | FFMQ |
| Räsänen (2016) | DASS-D | DASS-A | PSS | MHC-SF | FFMQ |
| Ritvo (2020) | PHQ-9 | BAI | PSS | - | FFMQ-SF |
| Rosen (2018) | - | - | - | - | MAAS |
| Russell (2018) | - | - | PSS | - | CAMS-R |
| Sagon (2018) | DASS-D | DASS-A | DASS-S | - | - |
| Sairanen (2019) | DASS-D | DASS-A | DASS-S | - | FFMQ |
| Scott (2018) | PHQ-9 | - | - | - | - |
| Segal (2020) | PHQ-9 | GAD-7 | - | - | - |
| Shore (2018) | - | - | - | - | FFMQ |
| Simister (2018) | CES-D | - | - | - | FFMQ |
| Stjernswärd (2017) | - | - | PSS | - | FFMQ |
| Tighe (2017) | PHQ-9 | - | - | - | - |
| Trompetter (2014)* | HADS-D | HADS-A | - | MHC-SF | FFMQ-SF |
|  | HADS-D | HADS-A | - | MHC-SF | FFMQ-SF |
| Van Emmerik (2018) | - | - | - | - | FFMQ |
| Viskovich (2019) | DASS-D | DASS-A | DASS-S | MHC-SF, SWLS | - |
| Wahbeh (2016) | CES-D | - | PSS | PANAS-PA | FFMQ |
| Wahbeh (2018) | CES-D | - | PSS | - | - |
| Walsh (2019) | - | - | PSS | - | - |
| Wolever (2012)* | CES-D | - | PSS | - | CAMS-R |
|  | CES-D | - | PSS | - | CAMS-R |
| Yang (2018) | - | - | PSS | GWBS | - |
| Yang (2019) | PHQ-9 | GAD-7 | - | - | FFMQ |
| Zernicke (2014)* | POMS-D | POMS-A | CSOSI | - | FFMQ |

*Note*.  BAI, Beck Anxiety Inventory; BDI-II, Beck Depression Inventory-II; CBT; CAMS-R, Cognitive and Affective Mindfulness Scale-Revised; CCAPS-34-A, Counseling Center Assessment of Psychological Symptoms – 34 – Anxiety subscale; CCAPS-34-D, Counseling Center Assessment of Psychological Symptoms – 34 – Depression subscale; CES-D, Center for Epidemiological Studies Depression Scale; CHIME, Comprehensive Inventory of Mindfulness Experience; CSOSI, Calgary Symptoms of Stress Inventory; DASS-A, Depression Anxiety and Stress Scale – Anxiety subscale; DASS-D, Depression Anxiety and Stress Scale – Depression subscale; DASS-S, Depression Anxiety and Stress Scale – Stress subscale; EPDS, Edinburgh Postnatal Depression Scale; F, female; FACT-G, Functional Assessment of Cancer Therapy – General Scale; FFMQ, Five Facet Mindfulness Questionnaire; FFMQ-SF, Five Facet Mindfulness Questionnaire – Short Form; FMI, Freiburg Mindfulness Inventory; FS, Flourishing Scale; GAD-7, Generalized Anxiety Disorder – 7; GWBS, General Well-Being Schedule; HADS-A, Hospital Anxiety and Depression Scale – Anxiety subscale; HADS-D, Hospital Anxiety and Depression Scale – Depression subscale; HAPA, Health Action Process Approach; LiSat-11, Life Satisfaction Questionnaire –11; MAAS, Mindful Attention Awareness Scale; MHC-SF, Mental Health Continuum-Short Form; MHI-A, Mental Health Inventory – Anxiety subscale; MHI-D, Mental Health Inventory – Depression subscale; MLQ-P, Meaning in Life Questionnaire – Presence subscale; PANAS-PA, Positive and Negative Affect Scale – Positive Affect subscale; PHQ-9, Patient Health Questionnaire - Depression Scale; PHQ-4-A, Patient Health Questionnaire - Anxiety subscale; PHQ-4-D, Patient Health Questionnaire - Depression subscale; POMS-A, Profile of Mood States – Anxiety Subscale; POMS-D, Profile of Mood States – Depression Subscale; PSS, Perceived Stress Scale; PSQ, Perceived Stress Questionnaire; PWBS, Psychological Well-Being Scale; QOLI, Quality of Life Inventory; SAS, Self-Rating Anxiety Scale; SDS, Self-Rating Depression Scale; SF-36-EW, RAND Corporation’s Medical Outcomes Study Short Form – 36 – Emotional Well-Being subscale; SWLS, Satisfaction With Life Scale; WBI, Well-Being Index; WEMWBS, Warwick Edinburgh Mental Well-Being Scale.

^a^We solely report outcome measures that were used in the meta-analysis.

*Studies included in the meta-analysis of Spijkerman et al. (2016).
