# Supplementary material for: New Evidence in the Booming Field of Online Mindfulness: An Updated Meta-analysis of Randomized Controlled Trials
Source: JMIR Ment Health. 2021 Jul 19;8(7):e28168. doi: 10.2196/28168 (PMC8329762; doi:10.2196/28168)
Supplement: Multimedia Appendix 2 [file mental_v8i7e28168_app2.docx]

Multimedia Appendix 2. Methodological quality of studies included in the meta-analysis.

| First author (year) | C1^a^ | C2^b^ | C3^c^ | C4^d^ | C5^e^ | C6^f^ | C7^g^ | Score |
| --- | --- | --- | --- | --- | --- | --- | --- | --- |
| Ahmad (2020) | Yes | Yes | No | Yes | Yes | Yes | Yes | 6 |
| Aikens (2014) | Yes | Yes | No | Yes | No | Yes | Yes | 5 |
| Allexandre (2016) | No | Yes | Yes | No | Yes | Yes | Yes | 5 |
| Barrett (2020) | Yes | Yes | Yes | Yes | Yes | Yes | Yes | 7 |
| Beshai (2020) | No | Yes | Yes | Yes | Yes | Yes | Yes | 6 |
| Boettcher (2014) | Yes | Yes | No | Yes | Unclear | Yes | Yes | 5 |
| Bostock (2018) | Yes | Yes | No | No | No | Yes | Yes | 4 |
| Bruggeman-Everts (2017) | Yes | Yes | No | Yes | Yes | Yes | No | 5 |
| Buhrman (2013) | Yes | Yes | No | Yes | Yes | No | No | 4 |
| Cavalera (2018) | Yes | Yes | No | Yes | Yes | Yes | Yes | 6 |
| Cavanagh (2013) | Yes | Yes | No | Yes | Unclear | Yes | Yes | 5 |
| Cavanagh (2018) | Yes | Yes | No | Yes | Yes | Yes | Yes | 6 |
| Champion (2018) | Yes | Yes | No | Yes | No | No | Yes | 4 |
| Chapoutot (2020) | Yes | Yes | Yes | Yes | Yes | Yes | Yes | 7 |
| Compen (2018) | Yes | Yes | Yes | Yes | Yes | Yes | Yes | 7 |
| Cox (2018) | No | Yes | No | No | No | Yes | Yes | 3 |
| De Wit (2020) | Yes | Yes | Yes | Yes | Yes | Yes | Yes | 7 |
| Dowd (2015) | Yes | Yes | No | Yes | Yes | Yes | No | 5 |
| El Morr (2020) | Yes | Yes | Unclear | Yes | Yes | Yes | Yes | 6 |
| Eriksson (2018) | Unclear | Yes | No | No | Unclear | Unclear | Yes | 2 |
| Eustis (2018) | Yes | Yes | No | No | Yes | Yes | Yes | 5 |
| Flett (2018) | Yes | Yes | No | No | No | Yes | Yes | 4 |
| Forbes (2020) | Yes | Yes | Yes | Yes | No | Yes | Yes | 6 |
| Gaigg (2020) | Yes | Yes | Yes | No | No | Yes | Yes | 5 |
| Gao (2021) | Yes | Yes | Yes | Yes | Yes | Yes | Yes | 7 |
| Garrisson (2018) | Yes | Yes | No | No | No | No | Yes | 3 |
| Gili (2020) | Yes | Yes | No | Yes | No | Yes | No | 4 |
| Glück (2011) | No | Yes | No | Yes | No | No | Yes | 3 |
| Gu (2018) | Yes | Yes | No | No | No | No | Yes | 3 |
| Hearn (2018) | Yes | Yes | No | No | Yes | Yes | No | 4 |
| Hearn (2019) | Yes | Yes | No | No | Yes | Yes | Yes | 5 |
| Henriksson (2016) | Yes | Yes | No | No | No | Yes | No | 3 |
| Hesser (2012) | Yes | Yes | No | No | Unclear | Yes | Yes | 4 |
| Hoffmann (2020) | Yes | Yes | Yes | Yes | Yes | Yes | Yes | 7 |
| Howells (2016) | Yes | Yes | No | No | No | Yes | Yes | 4 |
| Huberty (2019) | Yes | Yes | No | No | Yes | Yes | Yes | 5 |
| Ivtzan (2018) | Yes | Yes | No | Yes | No | Unclear | Yes | 4 |
| Jelinek (2020) | Yes | Yes | Yes | Yes | Yes | Yes | Yes | 7 |
| Khazaelli (2019) | Unclear | Unclear | Unclear | Unclear | No | Unclear | Yes | 1 |
| Kladnitski (2020) | Yes | Yes | Yes | Yes | Yes | Yes | Yes | 7 |
| Köhle (2021) | Yes | Yes | No | Yes | Yes | Yes | Yes | 6 |
| Krieger (2019) | Yes | Yes | No | Yes | No | No | Yes | 4 |
| Krusche (2018) | No | Yes | Yes | No | Yes | Yes | Yes | 5 |
| Kubo (2019) | Yes | Yes | No | No | No | No | Yes | 3 |
| Kubo (2020) | No | Yes | Yes | No | Yes | Yes | Yes | 5 |
| Kvillemo (2016) | Yes | Yes | Yes | Yes | No | Yes | Yes | 6 |
| Lappalainen (2015) | Yes | Yes | No | Yes | No | Yes | Yes | 5 |
| Lee (2018) | Unclear | Unclear | No | No | Yes | Yes | Yes | 3 |
| Levin (2014) | Yes | Yes | No | Yes | Yes | Yes | Yes | 7 |
| Levin (2016) | Yes | Yes | No | Yes | No | Yes | Yes | 5 |
| Levin (2017) | Yes | Yes | No | Yes | No | Yes | Yes | 5 |
| Levin (2019) | Yes | Yes | No | Yes | Yes | Yes | Yes | 6 |
| Levin (2020a) | Yes | Yes | No | No | No | Yes | Yes | 4 |
| Levin (2020b) | Yes | Yes | No | Yes | Yes | Yes | Yes | 6 |
| Lilly (2019) | Yes | Yes | No | Yes | No | Yes | Yes | 5 |
| Lin (2017) | Yes | Yes | No | Yes | Yes | Yes | Yes | 6 |
| Ly (2014) | Yes | Yes | No | Yes | Unclear | Yes | Yes | 5 |
| Ma (2018) | Yes | Yes | No | No | No | Yes | Yes | 4 |
| Mak (2015) | Yes | Yes | No | Yes | Yes | No | Yes | 5 |
| Mak (2017) | Yes | Yes | No | Yes | No | Unclear | Yes | 4 |
| Mak (2018) | Yes | Yes | No | Yes | No | Unclear | Yes | 4 |
| Messer (2019) | Yes | Yes | No | Yes | Yes | Yes | Yes | 6 |
| Milbury (2020) | Unclear | Yes | Unclear | No | No | Yes | Yes | 3 |
| Molander (2018) | Yes | Yes | No | Yes | Yes | Yes | Yes | 6 |
| Morledge (2013) | Yes | Yes | No | Yes | Yes | Yes | Yes | 7 |
| Nadler (2020) | Yes | Yes | No | No | Yes | Yes | Yes | 5 |
| Nguyen-Feng (2016) | Yes | Yes | No | Yes | No | Yes | Yes | 5 |
| Nguyen-Feng (2017) | Yes | Yes | No | No | No | Yes | Yes | 4 |
| Nissen (2020) | Yes | Yes | Yes | Yes | Yes | Yes | Yes | 7 |
| Noone (2018) | Yes | Yes | No | Yes | Yes | Yes | Yes | 6 |
| O’Driscoll (2019) | No | Yes | No | No | No | Yes | Yes | 3 |
| Potharst (2019) | Yes | Yes | No | Yes | Yes | Yes | Yes | 6 |
| Pots (2016) | Yes | Yes | No | Yes | Yes | Yes | Yes | 6 |
| Puzia (2020) | Yes | Yes | Unclear | No | No | Yes | Yes | 4 |
| Ritvo (2020) | Yes | Yes | No | Yes | Yes | Yes | Yes | 6 |
| Querstret (2017) | Yes | Yes | No | Yes | No | Yes | Yes | 5 |
| Räsänen (2016) | Yes | Yes | No | Yes | Yes | Yes | Yes | 6 |
| Rosen (2018) | Yes | Yes | Yes | No | Yes | Yes | Yes | 6 |
| Russell (2018) | Yes | Yes | No | No | No | Yes | Yes | 4 |
| Sagon (2018) | Yes | Yes | No | Yes | No | Yes | Yes | 5 |
| Sairanen (2019) | Yes | Yes | No | No | No | Yes | Yes | 4 |
| Scott (2018) | Yes | Yes | Yes | Yes | No | Yes | Yes | 6 |
| Segal (2020) | Yes | Yes | No | Yes | Yes | Yes | Yes | 6 |
| Shore (2018) | Yes | Yes | No | No | No | Yes | Yes | 4 |
| Simister (2018) | Yes | Yes | No | Yes | No | Yes | Yes | 5 |
| Stjernswärd (2017) | Yes | Yes | No | No | Yes | Yes | Yes | 5 |
| Tighe (2017) | Yes | No | Yes | Yes | No | Yes | Yes | 5 |
| Trompetter (2014) | Yes | Yes | No | Yes | Yes | Yes | No | 5 |
| Van Emmerik (2018) | Yes | Yes | No | Yes | Yes | Yes | Yes | 6 |
| Viskovich (2019) | Yes | Yes | Yes | Yes | Yes | Yes | Yes | 7 |
| Wahbeh (2016) | Yes | Yes | No | No | No | Yes | Yes | 4 |
| Wahbeh (2018) | Yes | Yes | No | No | No | Yes | Yes | 4 |
| Walsh (2019) | Yes | Yes | No | No | No | Yes | Yes | 4 |
| Wolever (2012) | Yes | Yes | No | Yes | Unclear | Unclear | Yes | 4 |
| Yang (2018) | Yes | Yes | No | No | No | Unclear | Yes | 3 |
| Yang (2019) | Yes | Yes | No | Yes | Yes | Yes | Yes | 6 |
| Zernicke (2014) | Yes | Yes | No | Yes | Yes | Yes | Yes | 6 |

*Note.* C, criterion.

^a^Adequate allocation sequence generation and allocation concealment.

^b^Blinding of main outcome assessments.

^c^Description of withdrawals/drop-outs.

^d^Intention-to-treat analysis is performed or there are no drop-outs.

^e^The sample size is based on an adequate power analysis.

^f^The groups are similar on prognostic indicators at baseline (and this was explicitly assessed) or adjustments were made to correct for baseline imbalance (using appropriate covariates).

^g^Diagnostic assessment was conducted by a professional, or there were no diagnostic assessments necessary for the recruitment.
