# Supplementary material for: New Evidence in the Booming Field of Online Mindfulness: An Updated Meta-analysis of Randomized Controlled Trials
Source: JMIR Ment Health. 2021 Jul 19;8(7):e28168. doi: 10.2196/28168 (PMC8329762; doi:10.2196/28168)
Supplement: Multimedia Appendix 3 [file mental_v8i7e28168_app3.docx]

Multimedia Appendix 3. Subgroup analyses

| Outcome | Criterion | Subgroup | *N*_comp_ | Hedges’ *g*^a^ | 95% CI | *I^2^* | *Z* | *P*^b^ |
| --- | --- | --- | --- | --- | --- | --- | --- | --- |

| Depression | Intervention type | |  |  |  |  |  |  |
| --- | --- | --- | --- | --- | --- | --- | --- | --- |
|  |  | MBSR | 10 | 0.16 | -.45 to .56 | 98.60 | 0.77 (ns) |  |
|  |  | MBCT | 14 | 0.32 | -.03 to .67 | 88.28 | 1.77 (ns) |  |
|  |  | ACT | 26 | 0.35 | 0.10 to .60 | 29.57 | 2.74** |  |
|  |  | MBI | 35 | 0.37 | .15 to .59 | 47.69 | 3.28** | .842 |
|  | Guidance | |  |  |  |  |  |  |
|  |  | With | 33 | 0.42 | .17 to .67 | 79.41 | 3.32*** |  |
|  |  | Without | 55 | 0.27 | .05 to .49 | 94.69 | 2.68** | .333 |
|  | Delivery mode | |  |  |  |  |  |  |
|  |  | App | 20 | 0.44 | .10 to .78 | 57.81 | 2.51** |  |
|  |  | Website | 66 | 0.31 | .12 to .49 | 94.74 | 3.26** | .518 |
|  | Population type | |  |  |  |  |  |  |
|  |  | Clinical | 45 | 0.44 | .22 to .65 | 75.36 | 4.02*** |  |
|  |  | Non-clinical | 44 | 0.23 | .01 to .44 | 95.58 | 2.09* | .180 |
|  | Symptoms | |  |  |  |  |  |  |
|  |  | No symptoms | 42 | 0.22 | .01 to .44 | 95.69 | 2.00* |  |
|  |  | Psychological | 24 | 0.51 | .22 to .80 | 83.34 | 3.47** |  |
|  |  | Physical | 23 | 0.35 | .06 to .65 | 0 | 2.35* | .293 |
|  | Type of control | Active | 40 | 0.26 | .02 to .47 | 96.06 | 2.13* |  |
|  |  | Inactive | 49 | 0.41 | .20 to .61 | 72.52 | 3.87*** | .302 |
| Anxiety | Intervention type | |  |  |  |  |  |  |
|  |  | MBSR | 8 | 0.28 | .05 to .51 | 78.39 | 2.42* |  |
|  |  | MBCT | 12 | 0.25 | .04 to .46 | 65.85 | 2.35* |  |
|  |  | ACT | 23 | 0.23 | .09 to .36 | 33.95 | 3.32** |  |
|  |  | MBI | 28 | 0.30 | .17 to .46 | 70.88 | 4.56*** | .881 |
|  | Guidance | |  |  |  |  |  |  |
|  |  | With | 27 | 0.30 | .19 to .42 | 61.31 | 5.02*** |  |
|  |  | Without | 46 | 0.22 | .13 to .32 | 63.43 | 4.58*** | .293 |
|  | Delivery mode | |  |  |  |  |  |  |
|  |  | App | 17 | 0.21 | .04 to .37 | 72.90 | 2.41* |  |
|  |  | Website | 55 | 0.27 | .18 to .35 | 61.85 | 6.03*** | .525 |
|  | Population type | |  |  |  |  |  |  |
|  |  | Clinical | 35 | 0.27 | .16 to .39 | 66.64 | 4.77*** |  |
|  |  | Non-clinical | 39 | 0.25 | .15 to .35 | 61.16 | 4.78*** | .817 |
|  | Symptoms | |  |  |  |  |  |  |
|  |  | No symptoms | 36 | 0.21 | .11 to .30 | 55.15 | 4.13*** |  |
|  |  | Psychological | 18 | 0.47 | .33 to .61 | 61.22 | 6.53*** |  |
|  |  | Physical | 20 | 0.16 | .03 to .30 | 54.24 | 2.38* | .008** |
|  | Type of control | |  |  |  |  |  |  |
|  |  | Active | 31 | 0.19 | .08 to .31 | 67.80 | 3.30** |  |
|  |  | Inactive | 43 | 0.31 | .21 to .41 | 59.63 | 6.09*** | .144 |
| Stress | Intervention type | |  |  |  |  |  |  |
|  |  | MBSR | 12 | 0.63 | .39 to .88 | 82.91 | 5.01*** |  |
|  |  | MBCT | 2 | - | - | - | - |  |
|  |  | ACT | 11 | 0.21 | -.05 to .47 | 39.20 | 1.56 (ns) |  |
|  |  | MBI | 27 | 0.43 | .26 to .60 | 82.24 | 5.09*** | .070 |
|  | Guidance | |  |  |  |  |  |  |
|  |  | With | 18 | 0.61 | .43 to .82 | 69.60 | 6.20*** |  |
|  |  | Without | 37 | 0.34 | .21 to .47 | 78.34 | 5.09*** | .017* |
|  | Delivery mode | |  |  |  |  |  |  |
|  |  | App | 9 | 0.38 | .10 to .66 | 88.79 | 2.67** |  |
|  |  | Website | 44 | 0.40 | .27 to .52 | 71.06 | 6.45*** | .918 |
|  | Population type | |  |  |  |  |  |  |
|  |  | Clinical | 11 | 0.57 | .30 to .84 | 64.04 | 4.13*** |  |
|  |  | Non-clinical | 45 | 0.41 | .28 to .53 | 80.83 | 6.36*** | .292 |
|  | Symptoms | No symptoms | 44 | 0.39 | .27 to .52 | 79.75 | 6.01*** |  |
|  |  | Psychological | 8 | 0.59 | .29 to .90 | 77.29 | 3.78** |  |
|  |  | Physical | 4 | - | - | - | - | .238 |
|  | Type of control | |  |  |  |  |  |  |
|  |  | Active | 18 | 0.15 | -.04 to .35 | 74.37 | 1.56 (ns) |  |
|  |  | Inactive | 38 | 0.56 | .43 to .69 | 77.82 | 8.35*** | .001** |
| Well-being | Intervention type | |  |  |  |  |  |  |
|  |  | MBSR | 8 | -0.01 | -1.65 to 0.62 | 99.36 | -.04 (ns) |  |
|  |  | MBCT | 5 | 0.01 | -.39 to .40 | 82.88 | .03 (*p*= .976) |  |
|  |  | ACT | 18 | 0.21 | .01 to .42 | 82.20 | 2.02* |  |
|  |  | MBI | 18 | 0.30 | .21 to .40 | 0 | 6.19*** | .791 |
|  | Guidance | |  |  |  |  |  |  |
|  |  | With | 20 | 0.17 | -.21 to .56 | 78.98 | 0.89 (ns) |  |
|  |  | Without | 32 | 0.23 | -.21 to .53 | 97.77 | 1.52 (ns) | .585 |
|  | Delivery mode | |  |  |  |  |  |  |
|  |  | App | 13 | 0.42 | -.13 to .98 | 83.71 | 1.49 (ns) |  |
|  |  | Website | 37 | 0.16 | -.17 to .48 | 97.40 | .92 (ns) | .413 |
|  | Population type | |  |  |  |  |  |  |
|  |  | Clinical | 21 | 0.21 | -.16 to .58 | 65.81 | 1.11 (ns) |  |
|  |  | Non-clinical | 31 | 0.21 | -.09 to .52 | 97.86 | 1.36 (ns) | .989 |
|  | Symptoms | |  |  |  |  |  |  |
|  |  | No symptoms | 31 | 0.21 | -.10 to .52 | 97.86 | 1.35 (ns) |  |
|  |  | Psychological | 9 | 0.25 | -.31 to .82 | 81.56 | 0.87 (ns) |  |
|  |  | Physical | 12 | 0.18 | -.32 to .67 | 15.81 | 0.70 (ns) | .980 |
|  | Type of control | |  |  |  |  |  |  |
|  |  | Active | 28 | 0.13 | -.19 to .45 | 97.99 | 0.81 (ns) |  |
|  |  | Inactive | 24 | 0.30 | -.04 to .65 | 67.13 | 1.72 (ns) | .474 |
| Mindfulness | Intervention type | |  |  |  |  |  |  |
|  |  | MBSR | 17 | 0.49 | .29 to .70 | 90.49 | 4.72*** |  |
|  |  | MBCT | 8 | 0.40 | .07 to .72 | 43.97 | 2.38* |  |
|  |  | ACT | 9 | 0.24 | -.05 to .50 | 41.98 | 1.61 (ns) |  |
|  |  | MBI | 35 | 0.40 | 0.24 to .55 | 75.81 | 4.72*** | .336 |
|  | Guidance | |  |  |  |  |  |  |
|  |  | With | 18 | 0.45 | .25 to .66 | 56.15 | 4.39*** |  |
|  |  | Without | 54 | 0.38 | .27 to .50 | 85.04 | 6.42*** | .563 |
|  | Delivery mode | |  |  |  |  |  |  |
|  |  | App | 16 | 0.36 | .13 to .57 | 88.32 | 3.15** |  |
|  |  | Website | 47 | 0.44 | .31 to .57 | 70.93 | 6.74*** | .528 |
|  | Population type | |  |  |  |  |  |  |
|  |  | Clinical | 25 | 0.29 | .11 to .47 | 68.85 | 3.22** |  |
|  |  | Non-clinical | 47 | 0.46 | .33 to .59 | 86.08 | 7.09*** | .123 |
|  | Symptoms | |  |  |  |  |  |  |
|  |  | No symptoms | 45 | 0.45 | .32 to .58 | 86.35 | 6.80*** |  |
|  |  | Psychological | 11 | 0.44 | .18 to .70 | 59.89 | 3.29** |  |
|  |  | Physical | 16 | 0.24 | .02 to .46 | 71.15 | 2.09* | .247 |
|  | Type of control | |  |  |  |  |  |  |
|  |  | Active | 25 | 0.19 | .03 to .34 | 73.24 | 2.43** |  |
|  |  | Inactive | 47 | 0.52 | .41 to .63 | 77.73 | 9.06*** | <.001*** |

*Note.* *N*_comp_, number of comparisons; CI, confidence interval.

^a^ Pre-post effects are reported.

^b^ The *P*-values in this column indicate whether the difference in Hedges’ g across subgroups is significant.

**P*<.05. ***P*<.01. ****P*<.001.
